# Long noncoding RNA UNC5B-AS1 suppresses cell proliferation by sponging miR-24-3p in glioblastoma multiforme

**DOI:** 10.1186/s12920-024-01851-5

**Published:** 2024-04-09

**Authors:** Ying Song, Baodong Chen, Huili Jiao, Li Yi

**Affiliations:** 1 Department of Neurology, Peking University Shenzhen Hospital, Shenzhen, 518036 China; 2 Department of Neurosurgery, Peking University Shenzhen Hospital, Shenzhen, 518036 China

**Keywords:** Glioblastoma multiforme, UNC5B-AS1, miR-24-3p, Survival, Cell proliferation

## Abstract

**Background:**

Glioblastoma multiforme (GBM) is the most common primary CNS tumor, characterized by high mortality and heterogeneity. However, the related lncRNA signatures and their target microRNA (miRNA) for GBM are still mostly unknown. Therefore, it is critical that we discover lncRNA markers in GBM and their biological activities.

**Materials and methods:**

GBM-related RNA-seq data were obtained from the Cancer Genome Atlas (TCGA) database. The “edger” R package was used for differently expressed lncRNAs (DELs) identification. Then, we forecasted prospective miRNAs that might bind to lncRNAs by Cytoscape software. Survival analysis of those miRNAs was examined by the starBase database, and Kyoto Encyclopedia of Genes and Genomes (KEGG) enrichment analysis of the miRNAs’ target genes was conducted by the Gene Set Enrichment Analysis (GSEA) database and R software. Moreover, the proliferative ability of unc-5 netrin receptor B antisense RNA 1 (UNC5B-AS1) cells was evaluated by Cell Counting Kit-8 (CCK-8) analysis. Mechanistically, the regulatory interaction between UNC5B-AS1 and miRNA in GBM biological processes was studied using CCK-8 analysis.

**Results:**

Our results indicated that overexpression of UNC5B-AS1 has been shown to suppress GBM cell growth. Mechanistically, miR-24-3p in GBM was able to alleviate the anti-oncogenic effects of UNC5B-AS1 on cell proliferation.

**Conclusion:**

The discovery of the novel UNC5B-AS1-miR-24-3p network suggests possible lncRNA and miRNA roles in the development of GBM, which may have significant ramifications for the analysis of clinical prognosis and the development of GBM medications.

**Supplementary Information:**

The online version contains supplementary material available at 10.1186/s12920-024-01851-5.

## Introduction

Glioblastoma multiforme (GBM), as the most prevalent and malignant category of central nervous system (CNS) tumours, has a very poor prognosis [1]. Every year, 3.19 cases of GBM are diagnosed per 100,000 people on average, and the median age at diagnosis is 64 [2]. To date, it is expected that the median overall survival for GBM is only 15 months after diagnosis [3]. Currently, surgical resection, adjuvant chemotherapy using temozolomide (TMZ) and radiation therapy make up the conventional treatment for GBM; however, surgery fails to cure GBM patients, and TMZ therapy always complies with the emergence of acquired resistance [4, 5]. Therefore, it is worthwhile to explore a better method for the diagnosis and therapeutic improvement of GBM. The 2021 World Health Organization Classification of Tumours of the Central Nervous System (2021 CNS WHO) advances the importance of molecular diagnostics in classifying CNS tumours [6]. However, based on our knowledge, few biomarkers have been discovered as effective treatment for GBM. Thus, it is urgent for us to digest GBM-related biomarkers and explore their biological functions in the tumorigenesis of GBM.

LncRNAs refer to RNA transcripts longer than 200 nucleotides that are not directly translated into proteins following transcription [7]. LncRNAs appear to play major roles in various cellular and biological functions of carcinogenesis and metastasis. Probably resulting from their critical roles in promoting or suppressing tumor growth, metastasis, differentiation, and different phrases of cell cycles [8, 9]. Interesting, lncRNAs are also considered competing endogenous RNAs (ceRNAs) to regulate RNA transcription by competing for the binding of microRNAs (miRNAs), which have attracted the attention of researchers [10]. Several studies have demonstrated the involvement of aberrant lncRNA levels in glioma progression via miRNA sponging, such as Linc01094, PCBP1-AS1 and MNX1-AS1 [11–13]. These molecular players provide novel insights into GBM and offer clues for developing anti-GBM therapies. However, the biological values and potential mechanisms of many lncRNAs in GBM have not been fully explored; finding and identifying new relevant lncRNAs and their potential mechanisms is crucial to improving the clinical outcome of GBM patients.

**Fig. 1:**
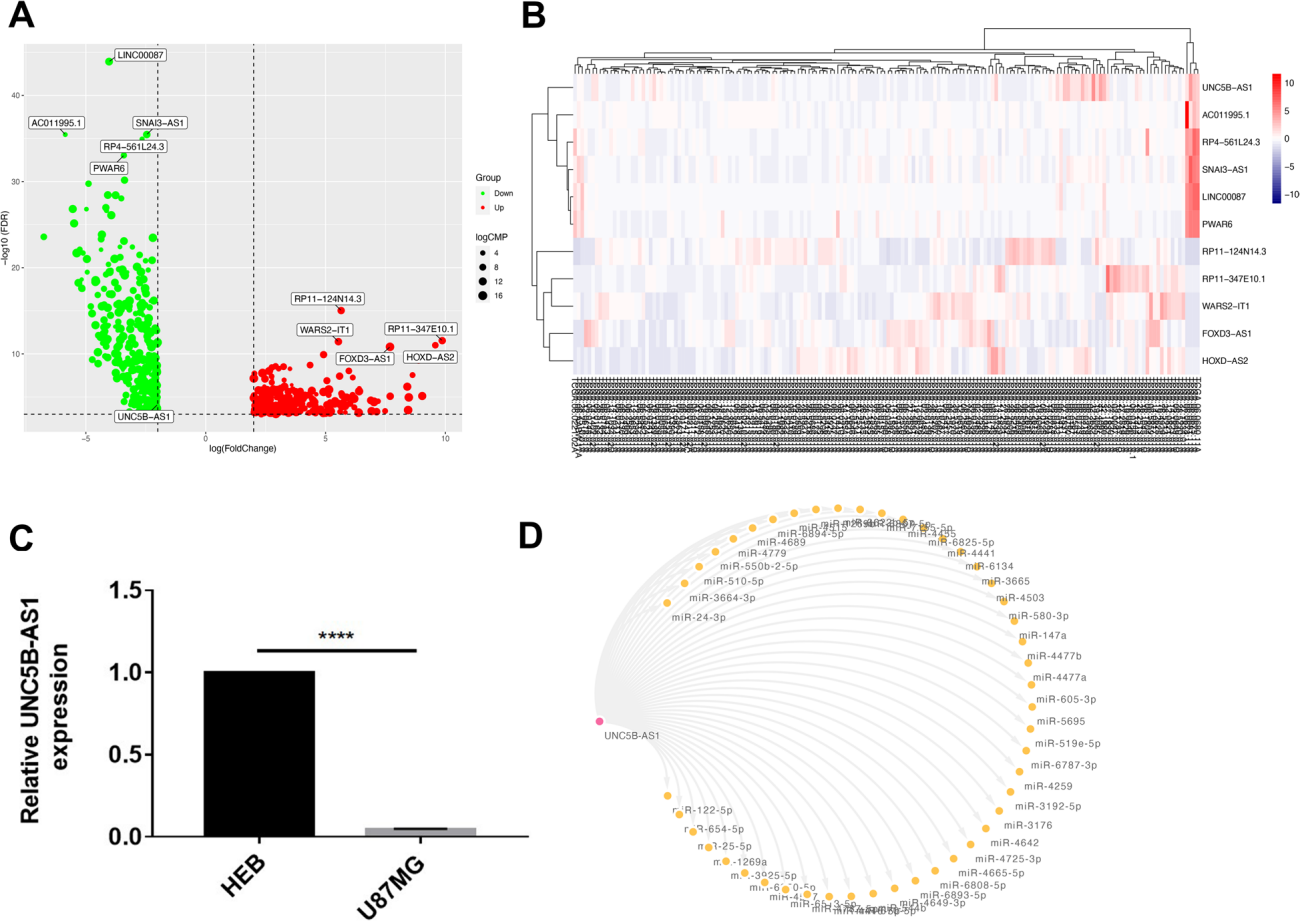
DELs identifying process and descriptions. **A** DEL volcano plot. LncRNAs that are upregulated are indicated by red dots, while lncRNAs that are downregulated are indicated by green dots. **B** Heatmap of DELs and UNC5B-AS1. Samples are represented on the horizontal axis, and DELs are represented on the vertical axis. **C** UNC5B-AS1 was expressed at lower levels in GBM. **D** A ceRNA regulation network of UNC5B-AS1 and downstream miRNAs. The pink and yellow circles indicate UNC5B-AS1 and miRNAs, respectively. Unpaired Student’s t test was utilized in **A**, ****p < 0.0001

This study aims to identify the potential lncRNA signatures as well as their potential mechanisms for the oncogenesis of GBM. Initially, 174 GBM patients’ RNA-seq data acquired from the TCGA database were used in this work (containing 5 normal brain samples and 169 GBM samples). UNC5B-AS1 was expressed at lower levels in GBM samples and cells than in normal groups based on our bioinformatic and experimental results. We also discovered that miR-24-3p, miR-654-5p and miR-580-3p can specifically target UNC5B-AS1 and involved in the prognosis of LGG. Those miRNAs and their target genes might regulate GBM tumorigenesis by regulating pathways in cancers, mitogen activated protein kinase (MAPK) signaling pathway and cell cycle progression. Moreover, the UNC5B-AS1-miR-24-3p regulatory interaction was verified in vitro. Finally, through mechanistic exploration, we found that lncRNA UNC5B-AS1 underlies the oncogenic function by interacting with miR-24-3p. The procedure for this study is displayed in A. Figure 1 in the Additional file 1.

## Materials and methods

### GBM dataset preparation

In this work, GBM sample RNA-seq data were collected from the publicly available TCGA database (https://www.cancer.gov/tcga), which contains 169 GBM samples and 5 normal brain samples.

### Identification of DELs in GBM

GENCODE database accesses users to annotate protein-coding and noncoding regions of human and mouse reference genomes, which draws on primary data as well as bioinformatic tools and analysis to enable the development of transcript structures and the identification of their functions [14]. We retained only the nonprotein-coding genes from the transcript sequences that were annotated in GENCODE. Then, we selected the lncRNA expression profiles across 174 GBM samples with a mean number of RPKM (reads per kilobase of exon model per million mapped reads) ≥ 0.1 [15]. Finally, lncRNAs that were expressed in at least 50% of the GBM samples were chosen for additional study. A bioconductor package called "edgeR" was used to examine differentially expressed lncRNAs between GBM and normal samples [16]. The thresholds were fixed at FDR < 0.001 and |log2FC|> 2. A volcano map and a heatmap plot were used to describe the results. All data was analyzed by R software.

### Identification of potential downstream miRNAs of UNC5B-AS1

The lncRNASNP2 database offers thorough resources on single nucleotide polymorphisms (SNPs) across both human and mouse lncRNAs and provides miRNA and lncRNA interaction outcomes integrated by the Pita, TargetScan and miRanda databases [17]. We used the lncRNASNP2 web tool to discover potential downstream miRNAs of UNC5B-AS1. Cystoscope, an open-source bioinformatics software platform, is far more effective for working with large networks and provides more flexibility in terms of networking, importing, and visualizing [18, 19]. By using Cytoscape software (version 3.7.0), a coexpression network of lncRNA-miRNAs was constructed.

### Three miRNAs’ targets enrichment analysis in KEGG pathways

The starBase database (https://starbase.sysu.edu.cn/index.php) is a free, accessible online tool that contains multidimensional sequencing data from 32 different kinds of cancer and enables investigation of RNA‒RNA interactions [20]. In this study, we examined the prognostic value of those miRNAs based on the miRNA's median value by using the starBase web tool. The GSEA web tool enable users to detect hallmark gene, chemical and genetic perturbations, KEGG and other gene sets analysis of mRNAs flexibility [21, 22]. We conducted enrichment analysis to explore the potential functions and mechanisms of the miRNAs’ targets based on GESA databases and visualized by the package ‘ggplot2’ built in R, we also conducted the KEGG pathway associated with GBM by Cytoscape software.

### Cell culture

All cells were purchased from BeNa Culture Collection (BNCC, China). The U87MG (BNCC; 338,150; China) and HEB cell lines (BNCC; 338,123; China) were cultured in 10-cm petri dishes containing penicillin‒streptomycin solution (Gibco; 2,257,223; Thermo Fisher Scientific) and 10% foetal bovine serum (FBS; 10,099–141; Gibco; Thermo Fisher Scientific) in Dulbecco's Modified Eagle Medium (DMEM; 8,121,356; Gibco; Thermo Fisher Scientific), which was maintained at 37 °C with 5% CO_2_, and the cells were then passaged every 2–3 days until they reached the logarithmic growth phase [23, 24].

### Quantitative real-time PCR (qRT-PCR)

We identified significant lncRNAs from those DELs for qRT‒PCR analysis. Total RNA was extracted from cells using a TRIzol kit (1 ml; QIAGEN; DP424; China) in line with the manufacturer’s guideline. Complementary DNA (cDNA) was synthesized by reverse transcription reagent containing gDNA Eraser (Cat# RR047A, TaKaRa) [25]. TB Green Premix (Cat# RR820A, TaKaRa) was used for qRT‒PCR, and the LightCycler® 96 Instrument (Roche, Switzerland) was used for analysis [26]. Based on the 2^−ΔΔCt^ method, we normalized the results and conducted our data analysis. The internal criteria for lncRNAs and miRNAs were β-actin and U6, respectively.

### Cell transfection

In this study, after 1 × 10^6^/well U87MG cells were plated onto 6-well plates, UNC5B-AS1 overexpression Lentivirus (sh-UNC5B-AS1) and negative control (sh-NC) were transfected into the U87MG cell line at an MOI = 5 for 72 h. Furthermore, the cells were incubated for 48 h in medium containing 2 µg/mL puromycin and then cultured in fresh DMEM. The Lentivirus vector GV367 was designed by Shanghai GeneChem Company (Shanghai, China). MiR-24-3p mimics and NC mimics were acquired from RiboBio Company (Guangzhou, China), and with the use of Lipofectamine 3000 (Invitrogen, L3000150, USA), cell transfection was accomplished [27]. Twenty-four hours before transfection, U87MG cells were seeded in 24-well plates until the density reached 50%, and the transfection concentration was 50 nM based on the manufacturer's guidelines. Then, the cells in each group were harvested for subsequent experiments.

### Cell proliferation assay

To assess cell proliferative capacity, the CCK-8 test was used. After transfection, U87MG cells were plated into 96‐well plates with 5 × 10^3^ cells per well and incubated for 24 h or 48 h in an incubator at 37 °C with 5% CO_2_ [4]. Subsequently, the cells received 10 µL of CCK-8 solution (Beyotime, C0039, China) and were then incubated for 2 h [28]. Finally, using a microplate reader, the optical density was calculated at a wavelength of 450 nm.

### Dual-luciferase assay

To identify probable binding sites between UNC5B-AS1 and miR-24-3p, the RNAhybrid (https://bibiserv.cebitec.uni-bielefeld.de/rnahybrid) web tool was utilized [29]. The artificially synthesized reporter plasmids were provided by GenePharma Company (GenePharma, Suzhou, China). A total of 5 × 10^4^ U87MG cells per well were plated into 96-well plates. UNC5B-AS1 WNT and MUT plasmids were separately transfected with miR-24-3p mimics or negative control by utilizing Lipofectamine 3000, and a dual luciferase assay solution (Promega, E1910, USA) was used to determine the luciferase activity after 48 h: 10ul LARII regent were added to detect the fluorescence value of firefly luciferase and 10ul stop&Go solution was added to test the Renilla fluorescence. The detected relative luciferase activity was normalized to the Renilla luciferase activity.

### Statistical analysis

GraphPad Prism 7.0 software was utilized to perform statistical analysis. The data are displayed as the mean ± S.D. To determine the statistical significance of a difference between two divisions, Student’s t test was used. We considered *p* values < 0.05 to be significant.

## Results

### Exploration of significant DELs and downstream miRNAs of UNC5B-AS1

A total of 622 differentially expressed lncRNAs were discovered (169 GBM tumour samples compared with 5 normal samples) with the thresholds | log 2 FC |> 2 and FDR < 0.001, including 252 upregulated lncRNAs and 370 downregulated lncRNAs (eTable 1 and eTable 2 in the Supplementary 1). The volcano map in Fig. 1A and heatmap in Fig. 1B display the distribution of the top 10 significant DELs and UNC5B-AS1.

In addition, qRT‒PCR was applied to estimate the expression of DELs. Considering that downregulated lncRNAs might serve as therapeutic targets or prognostic biomarkers in tumorigenesis, we focused on the identification of downregulated lncRNAs that orchestrate GBM progression and their phenotypic targets. Among them, lncRNA UNC5B-AS1, which was one of the prominently downregulated lncRNAs in GBM, was selected for further evaluation. As illustrated in Fig. 1C, the mRNA expression of UNC5B-AS1 in the U87MG cell line was downregulated compared to that in the HEB cell line, which is consistent with our bioinformatics analysis results above and had never been reported in GBM; therefore, we focused on UNC5B-AS1 for subsequent analysis.

**Fig. 2:**
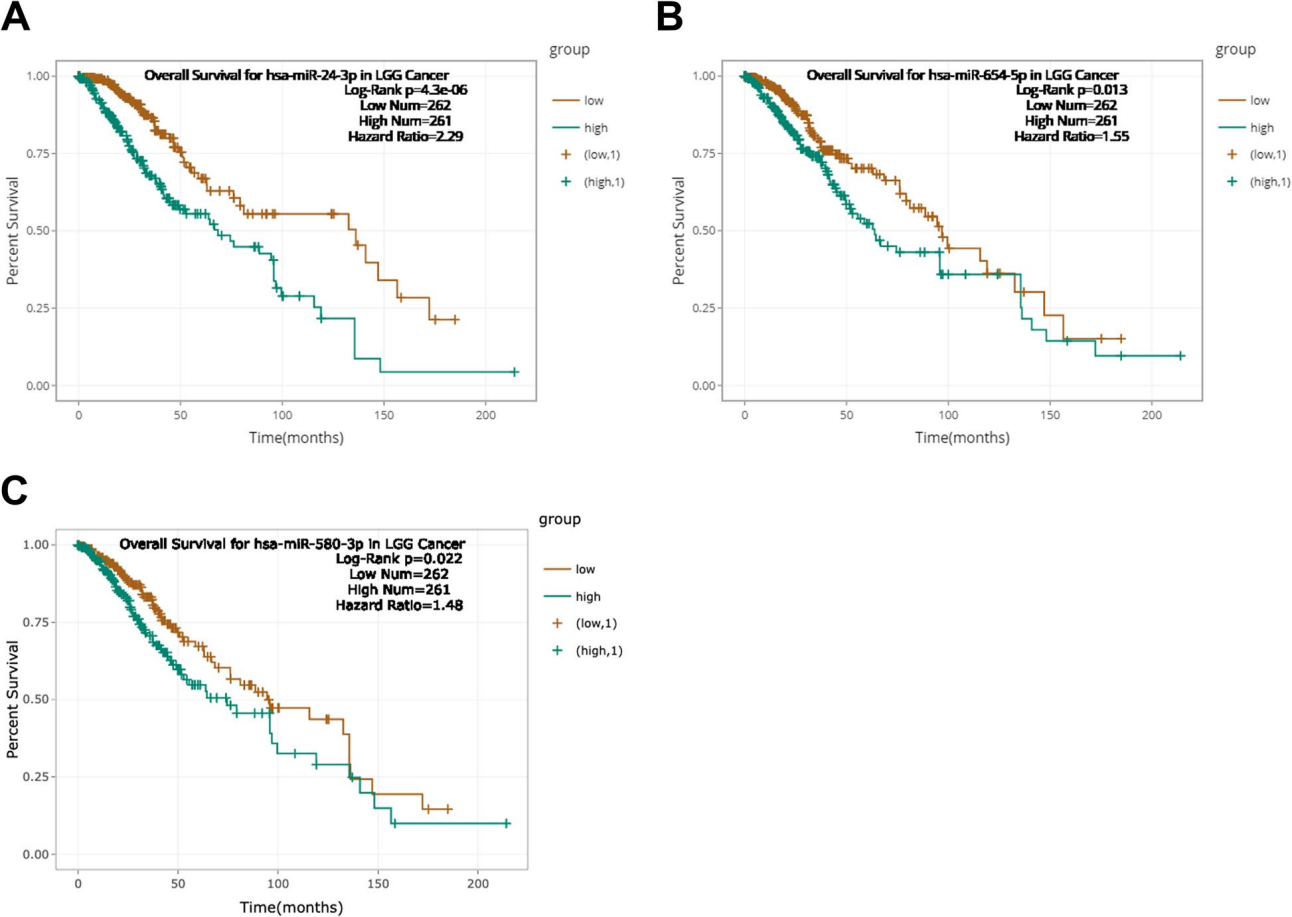
Survival analysis of downstream miRNAs of UNC5B-AS1 in LGG. **A** Overall survival of miR-24-3p. **B** Overall survival of miR-654-5p. **C** Overall survival of miR-580-3p. The horizontal axis represents time (months), and the vertical axis represents percent survival. The green colour represents the high expression group (*N* = 261), and the brown colour represents the low expression group (*N* = 262). *P* < 0.05

We further explored the potential miRNAs that bind to UNC5B-AS1 using the lncRNASNP2 web tool. A total of 47 miRNAs were identified and visualized by Cytoscape software (Fig. 1D). The starBase web tool was utilized to explore the prognosis of the downstream miRNAs of UNC5B-AS1. A total of 523 LGG patients were divided into two groups, including a high group (*N* = 261 patients) and a low group (*N* = 262 patients), based on the median miRNA expression value. Furthermore, the overall survival of these miRNAs was analyzed by plotting KM survival curves. Our results indicated that high expression of miR-24-3p, miR-654-5p and miR-580-3p was significantly associated with LGG patients’ prognosis, respectively (Fig. 2A, B and C). *P* < 0.05 was considered significant.

### KEGG enrichment analysis of the miRNAs’ targets

**Fig. 3:**
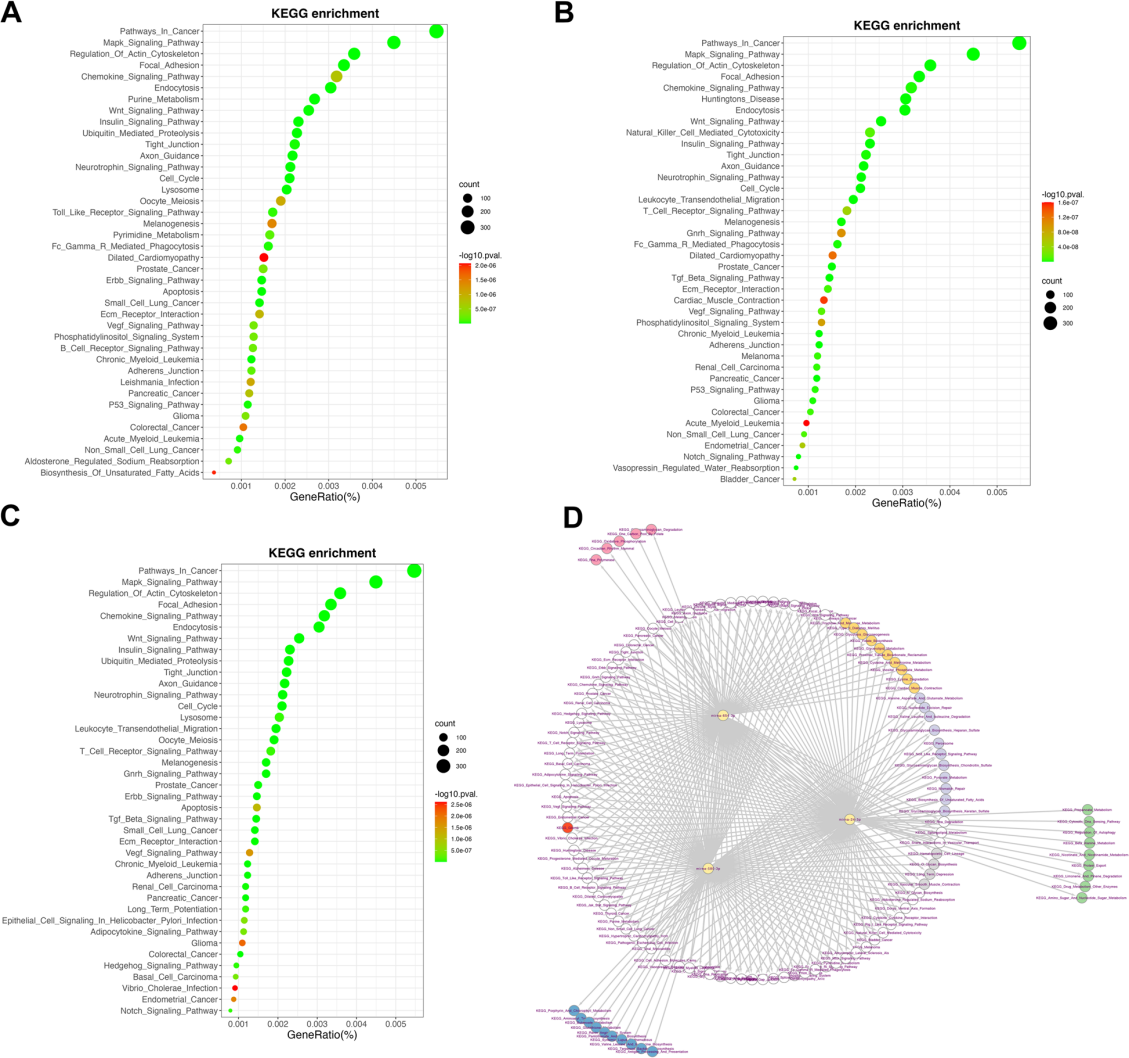
KEGG enrichment analysis of miRNAs’ targets. **A** Top 40 KEGG enrichment of the miR-24-3p target genes. **B** Top 40 KEGG enrichment of the miR-654-5p target genes. **C** Top 40 KEGG enrichment of the miR-580-3p target genes. The horizontal axis indicates GeneRatio (%), and the vertical axis indicates enrichment pathways. Count indicates gene numbers of each pathway. *P* < 0.05. **D** KEGG enrichment analysis of miRNAs’ target gene sets. The three yellow circles represent three miRNAs (miR-24-3p, miR-654-5p and miR-580-3p), respectively. The orange circles represent common enrichment pathways between miR-24-3p and miR-654-5p; The grey circles represent common enrichment pathways between miR-654-5p and miR-580-3p; The purple circles represent common enrichment pathways between miR-24-3p and miR-580-3p; The green, pink and blue circles represent independent enrichment pathways of miR-24-3p, miR-654-5p and miR-580-3p, respectively. The red circle represents glioma related pathways

KEGG enrichment analysis was carried out to determine the probable roles of miR-24-3p, miR-654-5p and miR-580-3p by the starBase database, respectively. Our findings showed that the three miRNAs' target genes were significantly correlated with the Pathways in Cancer, Regulation of Actin Cytoskeleton, Endocytosis and Glioma, suggesting potential roles for its coding proteins in various diseases (Fig. 3A-C and Tables 1, 2 and 3) and we visualized the regulatory network based on Cytoscape software (Fig. 3D). The results demonstrated that the three miRNAs might play pivotal role in administrating glioma tumorigenesis.

**Table 1:** The enrichment analysis of hsa-miR-24-3p targets in KEGG pathways

pathwayName	log10(pval)	log10(FDR)	Background GeneNum
KEGG_Pathways_In_Cancer	-21.11281	-18.8875	59,407
KEGG_Mapk_Signaling_Pathway	-19.03293	-17.10865	59,407
KEGG_Regulation_Of_Actin_Cytoskeleton	-15.08396	-13.46071	59,407
KEGG_Focal_Adhesion	-15.16291	-13.41472	59,407
KEGG_Endocytosis	-14.69126	-13.16492	59,407
KEGG_Cell_Cycle	-11.76121	-10.31405	59,407
KEGG_Neurotrophin_Signaling_Pathway	-11.68573	-10.30552	59,407
KEGG_Ubiquitin_Mediated_Proteolysis	-11.03921	-9.71699	59,407
KEGG_Axon_Guidance	-10.59925	-9.32818	59,407
KEGG_Apoptosis	-10.24157	-9.01626	59,407
KEGG_Insulin_Signaling_Pathway	-10.06958	-8.88567	59,407
KEGG_Chronic_Myeloid_Leukemia	-9.57462	-8.42849	59,407
KEGG_Lysosome	-9.4483	-8.33693	59,407
KEGG_Erbb_Signaling_Pathway	-9.29994	-8.22076	59,407
KEGG_Purine_Metabolism	-8.79863	-7.74942	59,407
KEGG_Small_Cell_Lung_Cancer	-8.62177	-7.60058	59,407
KEGG_Wnt_Signaling_Pathway	-8.47129	-7.47643	59,407
KEGG_Acute_Myeloid_Leukemia	-8.12584	-7.15581	59,407
KEGG_P53_Signaling_Pathway	-8.08432	-7.13777	59,407
KEGG_Tight_Junction	-7.98011	-7.05583	59,407
KEGG_Fc_Gamma_R_Mediated_Phagocytosis	-7.74824	-6.84515	59,407
KEGG_Non_Small_Cell_Lung_Cancer	-7.41331	-6.53042	59,407
KEGG_Toll_Like_Receptor_Signaling_Pathway	-7.36229	-6.49871	59,407
KEGG_Pyrimidine_Metabolism	-6.79865	-5.95355	59,407
KEGG_Adherens_Junction	-6.76612	-5.93875	59,407
KEGG_B_Cell_Receptor_Signaling_Pathway	-6.62292	-5.81259	59,407
KEGG_Prostate_Cancer	-6.53915	-5.79096	59,407
KEGG_Aldosterone_Regulated_Sodium_Reabsorption	-6.57985	-5.78591	59,407
KEGG_Vegf_Signaling_Pathway	-6.55313	-5.77498	59,407
KEGG_Phosphatidylinositol_Signaling_System	-6.55313	-5.77498	59,407
KEGG_Glioma	-6.47153	-5.73758	59,407
KEGG_Pancreatic_Cancer	-6.1078	-5.401	59,407
KEGG_Chemokine_Signaling_Pathway	-6.11785	-5.39769	59,407
KEGG_Ecm_Receptor_Interaction	-6.03402	-5.34019	59,407
KEGG_Leishmania_Infection	-5.97143	-5.30243	59,407
KEGG_Oocyte_Meiosis	-5.97933	-5.29808	59,407
KEGG_Melanogenesis	-5.85241	-5.19531	59,407
KEGG_Colorectal_Cancer	-5.80346	-5.15794	59,407
KEGG_Biosynthesis_Of_Unsaturated_Fatty_Acids	-5.69748	-5.06324	59,407
KEGG_Dilated_Cardiomyopathy	-5.6847	-5.06145	59,407
KEGG_Gap_Junction	-5.6847	-5.06145	59,407
KEGG_Amyotrophic_Lateral_Sclerosis_Als	-5.58365	-4.98159	59,407
KEGG_T_Cell_Receptor_Signaling_Pathway	-5.49914	-4.92705	59,407
KEGG_Vibrio_Cholerae_Infection	-5.50615	-4.9243	59,407
KEGG_Jak_Stat_Signaling_Pathway	-5.51153	-4.91969	59,407
KEGG_Lysine_Degradation	-5.38822	-4.82567	59,407
KEGG_Melanoma	-5.20493	-4.65172	59,407
KEGG_Glycosaminoglycan_Biosynthesis_Heparan_Sulfate	-5.15547	-4.6114	59,407
KEGG_Arrhythmogenic_Right_Ventricular_Cardiomyopathy_Arvc	-5.02663	-4.49152	59,407
KEGG_Snare_Interactions_In_Vesicular_Transport	-4.94959	-4.42325	59,407
KEGG_Mtor_Signaling_Pathway	-4.76369	-4.24595	59,407
KEGG_Endometrial_Cancer	-4.76369	-4.24595	59,407
KEGG_Epithelial_Cell_Signaling_In_Helicobacter_Pylori_Infection	-4.58148	-4.08045	59,407
KEGG_Hypertrophic_Cardiomyopathy_Hcm	-4.54326	-4.05035	59,407
KEGG_Alzheimers_Disease	-4.51789	-4.04077	59,407
KEGG_Ppar_Signaling_Pathway	-4.52489	-4.03994	59,407
KEGG_Amino_Sugar_And_Nucleotide_Sugar_Metabolism	-4.4575	-3.98807	59,407
KEGG_Progesterone_Mediated_Oocyte_Maturation	-4.44507	-3.98319	59,407
KEGG_Tgf_Beta_Signaling_Pathway	-4.39711	-3.94265	59,407
KEGG_Prion_Diseases	-4.24517	-3.79802	59,407
KEGG_Glycerophospholipid_Metabolism	-4.10728	-3.6673	59,407
KEGG_Cytokine_Cytokine_Receptor_Interaction	-3.95157	-3.51865	59,407
KEGG_Adipocytokine_Signaling_Pathway	-3.86767	-3.4417	59,407
KEGG_Inositol_Phosphate_Metabolism	-3.80034	-3.38121	59,407
KEGG_Gnrh_Signaling_Pathway	-3.75616	-3.34377	59,407
KEGG_Basal_Cell_Carcinoma	-3.7432	-3.33743	59,407
KEGG_Bladder_Cancer	-3.71875	-3.33229	59,407
KEGG_Long_Term_Potentiation	-3.7193	-3.32006	59,407
KEGG_Renal_Cell_Carcinoma	-3.7193	-3.32006	59,407
KEGG_Calcium_Signaling_Pathway	-3.62147	-3.24126	59,407
KEGG_Vasopressin_Regulated_Water_Reabsorption	-3.58826	-3.21421	59,407
KEGG_Alanine_Aspartate_And_Glutamate_Metabolism	-3.54909	-3.18111	59,407
KEGG_Huntingtons_Disease	-3.51732	-3.15533	59,407
KEGG_Propanoate_Metabolism	-3.4727	-3.11662	59,407
KEGG_Notch_Signaling_Pathway	-3.40596	-3.05571	59,407
KEGG_Dna_Replication	-3.25976	-2.91526	59,407
KEGG_Cytosolic_Dna_Sensing_Pathway	-2.9846	-2.64578	59,407
KEGG_Spliceosome	-2.90632	-2.5731	59,407
KEGG_Thyroid_Cancer	-2.86438	-2.5367	59,407
KEGG_Rna_Degradation	-2.80255	-2.48033	59,407
KEGG_Cell_Adhesion_Molecules_Cams	-2.74485	-2.42802	59,407
KEGG_Nod_Like_Receptor_Signaling_Pathway	-2.67632	-2.37009	59,407
KEGG_Glycolysis_Gluconeogenesis	-2.67632	-2.37009	59,407
KEGG_Leukocyte_Transendothelial_Migration	-2.67684	-2.36535	59,407
KEGG_Natural_Killer_Cell_Mediated_Cytotoxicity	-2.64304	-2.34715	59,407
KEGG_Type_Ii_Diabetes_Mellitus	-2.63607	-2.34526	59,407
KEGG_Glycerolipid_Metabolism	-2.54266	-2.26674	59,407
KEGG_Fructose_And_Mannose_Metabolism	-2.54663	-2.26084	59,407
KEGG_Cysteine_And_Methionine_Metabolism	-2.54663	-2.26084	59,407
KEGG_Glycosaminoglycan_Biosynthesis_Chondroitin_Sulfate	-2.46628	-2.20001	59,407
KEGG_Folate_Biosynthesis	-2.46965	-2.19859	59,407
KEGG_Proximal_Tubule_Bicarbonate_Reclamation	-2.39331	-2.13179	59,407
KEGG_Rig_I_Like_Receptor_Signaling_Pathway	-2.34181	-2.08498	59,407
KEGG_Dorso_Ventral_Axis_Formation	-2.32412	-2.07194	59,407
KEGG_Pathogenic_Escherichia_Coli_Infection	-2.25118	-2.00359	59,407
KEGG_Fc_Epsilon_Ri_Signaling_Pathway	-2.08968	-1.84664	59,407
KEGG_Glycosaminoglycan_Biosynthesis_Keratan_Sulfate	-2.06678	-1.82824	59,407
KEGG_Valine_Leucine_And_Isoleucine_Degradation	-2.05867	-1.82459	59,407
KEGG_Nucleotide_Excision_Repair	-2.05867	-1.82459	59,407
KEGG_N_Glycan_Biosynthesis	-1.97812	-1.75282	59,407
KEGG_Viral_Myocarditis	-1.79262	-1.57591	59,407
KEGG_Drug_Metabolism_Other_Enzymes	-1.79548	-1.57449	59,407
KEGG_Regulation_Of_Autophagy	-1.74064	-1.52817	59,407
KEGG_Hedgehog_Signaling_Pathway	-1.63539	-1.42711	59,407
KEGG_Beta_Alanine_Metabolism	-1.60259	-1.39847	59,407
KEGG_Sphingolipid_Metabolism	-1.58384	-1.38792	59,407
KEGG_Peroxisome	-1.58428	-1.38428	59,407
KEGG_Cardiac_Muscle_Contraction	-1.5604	-1.36852	59,407
KEGG_Pyruvate_Metabolism	-1.54789	-1.36397	59,407
KEGG_Mismatch_Repair	-1.55116	-1.36328	59,407
KEGG_Nicotinate_And_Nicotinamide_Metabolism	-1.50243	-1.32244	59,407
KEGG_Protein_Export	-1.50243	-1.32244	59,407
KEGG_Limonene_And_Pinene_Degradation	-1.48791	-1.31568	59,407

**Table 2:** The enrichment analysis of hsa-miR-654-5p targets in KEGG pathways

pathwayName	log10(pval)	log10(FDR)	Background GeneNum
KEGG_Pathways_In_Cancer	-26.22694	-24.31049	59,407
KEGG_Regulation_Of_Actin_Cytoskeleton	-26.50621	-24.28873	59,407
KEGG_Endocytosis	-24.8089	-23.06853	59,407
KEGG_Focal_Adhesion	-21.17816	-19.56273	59,407
KEGG_Mapk_Signaling_Pathway	-17.87455	-16.35603	59,407
KEGG_Axon_Guidance	-17.2517	-15.81237	59,407
KEGG_Adherens_Junction	-16.57521	-15.20283	59,407
KEGG_Neurotrophin_Signaling_Pathway	-15.59957	-14.28518	59,407
KEGG_Wnt_Signaling_Pathway	-15.2388	-13.97556	59,407
KEGG_Chronic_Myeloid_Leukemia	-13.20203	-11.98455	59,407
KEGG_Prostate_Cancer	-12.42598	-11.24989	59,407
KEGG_Insulin_Signaling_Pathway	-11.94704	-10.80873	59,407
KEGG_Leukocyte_Transendothelial_Migration	-11.83417	-10.73063	59,407
KEGG_Cell_Cycle	-11.12653	-10.05518	59,407
KEGG_Tgf_Beta_Signaling_Pathway	-10.76756	-9.72617	59,407
KEGG_Huntingtons_Disease	-10.64381	-9.63045	59,407
KEGG_Pancreatic_Cancer	-10.40562	-9.44341	59,407
KEGG_Vasopressin_Regulated_Water_Reabsorption	-10.4158	-9.42877	59,407
KEGG_Notch_Signaling_Pathway	-9.98088	-9.04215	59,407
KEGG_Melanogenesis	-9.48592	-8.56947	59,407
KEGG_Fc_Gamma_R_Mediated_Phagocytosis	-8.99858	-8.12351	59,407
KEGG_Tight_Junction	-9.00325	-8.10799	59,407
KEGG_Glioma	-8.92467	-8.06891	59,407
KEGG_Chemokine_Signaling_Pathway	-8.83754	-8.00026	59,407
KEGG_P53_Signaling_Pathway	-8.63259	-7.81305	59,407
KEGG_Renal_Cell_Carcinoma	-8.44686	-7.64435	59,407
KEGG_Melanoma	-8.35652	-7.5704	59,407
KEGG_Colorectal_Cancer	-8.24124	-7.47091	59,407
KEGG_Ecm_Receptor_Interaction	-8.1772	-7.42211	59,407
KEGG_Non_Small_Cell_Lung_Cancer	-8.06057	-7.3202	59,407
KEGG_Natural_Killer_Cell_Mediated_ Cytotoxicity	-7.94641	-7.22028	59,407
KEGG_Vegf_Signaling_Pathway	-7.92801	-7.21567	59,407
KEGG_T_Cell_Receptor_Signaling_Pathway	-7.34867	-6.6497	59,407
KEGG_Bladder_Cancer	-7.32659	-6.64059	59,407
KEGG_Endometrial_Cancer	-7.26687	-6.59346	59,407
KEGG_Phosphatidylinositol_Signaling_System	-7.04501	-6.38382	59,407
KEGG_Gnrh_Signaling_Pathway	-6.99537	-6.34609	59,407
KEGG_Dilated_Cardiomyopathy	-6.89849	-6.26079	59,407
KEGG_Cardiac_Muscle_Contraction	-6.82517	-6.19875	59,407
KEGG_Acute_Myeloid_Leukemia	-6.79462	-6.17919	59,407
KEGG_Calcium_Signaling_Pathway	-6.6733	-6.0686	59,407
KEGG_Ubiquitin_Mediated_Proteolysis	-6.6099	-6.02589	59,407
KEGG_Lysosome	-6.61046	-6.01623	59,407
KEGG_Aldosterone_Regulated_Sodium_Reabsorption	-6.29189	-5.71786	59,407
KEGG_Lysine_Degradation	-6.08966	-5.52539	59,407
KEGG_Epithelial_Cell_Signaling_In_Helicobacter_Pylori_Infection	-5.91962	-5.36489	59,407
KEGG_Long_Term_Potentiation	-5.77997	-5.23458	59,407
KEGG_Small_Cell_Lung_Cancer	-5.68638	-5.15014	59,407
KEGG_Cell_Adhesion_Molecules_Cams	-5.35307	-4.82578	59,407
KEGG_Alzheimers_Disease	-5.29015	-4.77164	59,407
KEGG_Vibrio_Cholerae_Infection	-5.22865	-4.71873	59,407
KEGG_Inositol_Phosphate_Metabolism	-5.22865	-4.71873	59,407
KEGG_Basal_Cell_Carcinoma	-5.1538	-4.6606	59,407
KEGG_Cysteine_And_Methionine_Metabolism	-5.10466	-4.61957	59,407
KEGG_Hypertrophic_Cardiomyopathy_Hcm	-4.98298	-4.50586	59,407
KEGG_Viral_Myocarditis	-4.97084	-4.50154	59,407
KEGG_Progesterone_Mediated_Oocyte_Maturation	-4.87485	-4.41324	59,407
KEGG_Vascular_Smooth_Muscle_Contraction	-4.8226	-4.36854	59,407
KEGG_Arrhythmogenic_Right_Ventricular_Cardiomyopathy_Arvc	-4.73442	-4.28779	59,407
KEGG_Snare_Interactions_In_Vesicular_Transport	-4.72257	-4.28323	59,407
KEGG_B_Cell_Receptor_Signaling_Pathway	-4.67791	-4.24576	59,407
KEGG_Gap_Junction	-4.61865	-4.19356	59,407
KEGG_Thyroid_Cancer	-4.61084	-4.1927	59,407
KEGG_Mtor_Signaling_Pathway	-4.51653	-4.10523	59,407
KEGG_Jak_Stat_Signaling_Pathway	-4.50802	-4.10345	59,407
KEGG_Fc_Epsilon_Ri_Signaling_Pathway	-4.46139	-4.06345	59,407
KEGG_Cytokine_Cytokine_Receptor_Interaction	-4.43988	-4.04847	59,407
KEGG_Purine_Metabolism	-4.37248	-3.9875	59,407
KEGG_Oocyte_Meiosis	-4.26165	-3.88301	59,407
KEGG_Dorso_Ventral_Axis_Formation	-4.11641	-3.74402	59,407
KEGG_Erbb_Signaling_Pathway	-4.06893	-3.7027	59,407
KEGG_Glycerophospholipid_Metabolism	-3.84958	-3.48943	59,407
KEGG_Pathogenic_Escherichia_Coli_Infection	-3.4755	-3.12134	59,407
KEGG_Toll_Like_Receptor_Signaling_Pathway	-3.44997	-3.10172	59,407
KEGG_N_Glycan_Biosynthesis	-3.27663	-2.93421	59,407
KEGG_Circadian_Rhythm_Mammal	-3.26639	-2.92972	59,407
KEGG_Proximal_Tubule_Bicarbonate_Reclamation	-3.20591	-2.87491	59,407
KEGG_Spliceosome	-3.18207	-2.85668	59,407
KEGG_Prion_Diseases	-3.16256	-2.8427	59,407
KEGG_Pyrimidine_Metabolism	-2.99936	-2.68497	59,407
KEGG_Adipocytokine_Signaling_Pathway	-2.94472	-2.63572	59,407
KEGG_Sphingolipid_Metabolism	-2.90548	-2.60181	59,407
KEGG_Long_Term_Depression	-2.8198	-2.5214	59,407
KEGG_Apoptosis	-2.79534	-2.50213	59,407
KEGG_Rna_Polymerase	-2.72566	-2.4376	59,407
KEGG_Glycerolipid_Metabolism	-2.38819	-2.10521	59,407
KEGG_Folate_Biosynthesis	-2.38076	-2.10279	59,407
KEGG_Amyotrophic_Lateral_Sclerosis_Als	-2.21857	-1.94557	59,407
KEGG_Rig_I_Like_Receptor_Signaling_Pathway	-2.17361	-1.90551	59,407
KEGG_Leishmania_Infection	-2.14079	-1.87755	59,407
KEGG_Hedgehog_Signaling_Pathway	-2.10221	-1.84377	59,407
KEGG_Oxidative_Phosphorylation	-2.06169	-1.80799	59,407
KEGG_Rna_Degradation	-1.994	-1.745	59,407
KEGG_Glycolysis_Gluconeogenesis	-1.89309	-1.64873	59,407
KEGG_O_Glycan_Biosynthesis	-1.8658	-1.62604	59,407
KEGG_One_Carbon_Pool_By_Folate	-1.82635	-1.59114	59,407
KEGG_Type_Ii_Diabetes_Mellitus	-1.81465	-1.58394	59,407
KEGG_Hematopoietic_Cell_Lineage	-1.71548	-1.48922	59,407
KEGG_Fructose_And_Mannose_Metabolism	-1.67983	-1.46235	59,407
KEGG_Ppar_Signaling_Pathway	-1.68211	-1.46026	59,407
KEGG_Dna_Replication	-1.59714	-1.38398	59,407
KEGG_Glycosaminoglycan_Degradation	-1.57507	-1.36619	59,407

**Table 3:** The enrichment analysis of hsa-miR-580-3p targets in KEGG pathways

pathwayName	log10(pval)	log10(FDR)	Background GeneNum
KEGG_Pathways_In_Cancer	-40.54821	-38.28814	59,407
KEGG_Wnt_Signaling_Pathway	-27.67344	-25.7144	59,407
KEGG_Focal_Adhesion	-24.60436	-22.82141	59,407
KEGG_Endocytosis	-23.32816	-21.67015	59,407
KEGG_Mapk_Signaling_Pathway	-21.04027	-19.47917	59,407
KEGG_Regulation_Of_Actin_Cytoskeleton	-17.01922	-15.5373	59,407
KEGG_Neurotrophin_Signaling_Pathway	-14.65132	-13.23635	59,407
KEGG_Tgf_Beta_Signaling_Pathway	-14.58431	-13.22733	59,407
KEGG_Adherens_Junction	-13.94784	-12.64201	59,407
KEGG_Small_Cell_Lung_Cancer	-13.73586	-12.47579	59,407
KEGG_Ubiquitin_Mediated_Proteolysis	-12.97806	-11.75938	59,407
KEGG_Insulin_Signaling_Pathway	-11.93987	-10.75898	59,407
KEGG_Chronic_Myeloid_Leukemia	-11.76125	-10.61512	59,407
KEGG_Leukocyte_Transendothelial_Migration	-11.72507	-10.61113	59,407
KEGG_Axon_Guidance	-11.638	-10.55402	59,407
KEGG_Melanogenesis	-11.08548	-10.02953	59,407
KEGG_Cell_Cycle	-10.17991	-9.15028	59,407
KEGG_Oocyte_Meiosis	-10.15229	-9.14749	59,407
KEGG_Pancreatic_Cancer	-9.99243	-9.01111	59,407
KEGG_Colorectal_Cancer	-9.78548	-8.82644	59,407
KEGG_Tight_Junction	-9.72192	-8.78407	59,407
KEGG_Ecm_Receptor_Interaction	-9.68272	-8.76508	59,407
KEGG_Erbb_Signaling_Pathway	-9.43073	-8.53239	59,407
KEGG_Gnrh_Signaling_Pathway	-9.25726	-8.3774	59,407
KEGG_Chemokine_Signaling_Pathway	-8.93285	-8.07072	59,407
KEGG_Prostate_Cancer	-8.36102	-7.51592	59,407
KEGG_Renal_Cell_Carcinoma	-8.02063	-7.19192	59,407
KEGG_Hedgehog_Signaling_Pathway	-7.31869	-6.50578	59,407
KEGG_Lysosome	-7.17663	-6.39368	59,407
KEGG_Notch_Signaling_Pathway	-7.18224	-6.38457	59,407
KEGG_T_Cell_Receptor_Signaling_Pathway	-7.12151	-6.3528	59,407
KEGG_Long_Term_Potentiation	-7.08984	-6.33492	59,407
KEGG_Basal_Cell_Carcinoma	-6.43594	-5.69439	59,407
KEGG_Adipocytokine_Signaling_Pathway	-6.41309	-5.6845	59,407
KEGG_Epithelial_Cell_Signaling_In_Helicobacter_Pylori_Infection	-6.34016	-5.62415	59,407
KEGG_Apoptosis	-5.95083	-5.24707	59,407
KEGG_Vegf_Signaling_Pathway	-5.80177	-5.1099	59,407
KEGG_Endometrial_Cancer	-5.73999	-5.05971	59,407
KEGG_Glioma	-5.67512	-5.00611	59,407
KEGG_Vibrio_Cholerae_Infection	-5.58268	-4.92467	59,407
KEGG_Huntingtons_Disease	-5.27488	-4.63806	59,407
KEGG_Progesterone_Mediated_Oocyte_Maturation	-5.27653	-4.62924	59,407
KEGG_Alzheimers_Disease	-5.22026	-4.59366	59,407
KEGG_Toll_Like_Receptor_Signaling_Pathway	-5.16113	-4.54452	59,407
KEGG_B_Cell_Receptor_Signaling_Pathway	-5.0498	-4.44294	59,407
KEGG_Dilated_Cardiomyopathy	-5.01482	-4.4175	59,407
KEGG_Jak_Stat_Signaling_Pathway	-4.99486	-4.40688	59,407
KEGG_Thyroid_Cancer	-4.86908	-4.29025	59,407
KEGG_Purine_Metabolism	-4.85484	-4.28497	59,407
KEGG_Non_Small_Cell_Lung_Cancer	-4.69404	-4.13294	59,407
KEGG_Hypertrophic_Cardiomyopathy_Hcm	-4.62199	-4.06949	59,407
KEGG_Biosynthesis_Of_Unsaturated_Fatty_Acids	-4.57403	-4.02996	59,407
KEGG_Pathogenic_Escherichia_Coli_Infection	-4.5625	-4.0267	59,407
KEGG_Viral_Myocarditis	-4.54225	-4.02254	59,407
KEGG_Cell_Adhesion_Molecules_Cams	-4.54416	-4.01648	59,407
KEGG_Vasopressin_Regulated_Water_Reabsorption	-4.51871	-4.00682	59,407
KEGG_Acute_Myeloid_Leukemia	-4.49894	-3.99474	59,407
KEGG_Glycosaminoglycan_Biosynthesis_Keratan_Sulfate	-4.35582	-3.85918	59,407
KEGG_Calcium_Signaling_Pathway	-4.25178	-3.76256	59,407
KEGG_Dna_Replication	-4.21647	-3.73455	59,407
KEGG_Glycerophospholipid_Metabolism	-4.17862	-3.70388	59,407
KEGG_P53_Signaling_Pathway	-3.8819	-3.41422	59,407
KEGG_Ppar_Signaling_Pathway	-3.83223	-3.3715	59,407
KEGG_Antigen_Processing_And_Presentation	-3.68673	-3.23957	59,407
KEGG_Leishmania_Infection	-3.68867	-3.23478	59,407
KEGG_Gap_Junction	-3.60604	-3.16551	59,407
KEGG_Arrhythmogenic_Right_Ventricular_Cardiomyopathy_Arvc	-3.59723	-3.16324	59,407
KEGG_Spliceosome	-3.53788	-3.11032	59,407
KEGG_Phosphatidylinositol_Signaling_System	-3.50899	-3.08777	59,407
KEGG_Glycosaminoglycan_Biosynthesis_Chondroitin_Sulfate	-3.485	-3.07003	59,407
KEGG_Nod_Like_Receptor_Signaling_Pathway	-3.43466	-3.02585	59,407
KEGG_Peroxisome	-3.42376	-3.02102	59,407
KEGG_Prion_Diseases	-3.37389	-2.98887	59,407
KEGG_Fc_Gamma_R_Mediated_Phagocytosis	-3.37776	-2.98692	59,407
KEGG_Fc_Epsilon_Ri_Signaling_Pathway	-3.38222	-2.98547	59,407
KEGG_Vascular_Smooth_Muscle_Contraction	-3.33647	-2.95721	59,407
KEGG_Pyrimidine_Metabolism	-3.30593	-2.93235	59,407
KEGG_Mtor_Signaling_Pathway	-3.18355	-2.81558	59,407
KEGG_Terpenoid_Backbone_Biosynthesis	-3.15743	-2.79498	59,407
KEGG_Glycosaminoglycan_Biosynthesis_Heparan_Sulfate	-3.12848	-2.77689	59,407
KEGG_Amyotrophic_Lateral_Sclerosis_Als	-3.13274	-2.77576	59,407
KEGG_Long_Term_Depression	-3.0756	-2.72935	59,407
KEGG_Melanoma	-3.03452	-2.69353	59,407
KEGG_Bladder_Cancer	-2.93799	-2.6022	59,407
KEGG_Natural_Killer_Cell_Mediated_Cytotoxicity	-2.70975	-2.3791	59,407
KEGG_Valine_Leucine_And_Isoleucine_Biosynthesis	-2.49408	-2.16851	59,407
KEGG_Hematopoietic_Cell_Lineage	-2.46756	-2.14701	59,407
KEGG_Rig_I_Like_Receptor_Signaling_Pathway	-2.38854	-2.07295	59,407
KEGG_Cytokine_Cytokine_Receptor_Interaction	-2.37838	-2.0677	59,407
KEGG_Dorso_Ventral_Axis_Formation	-2.35472	-2.04889	59,407
KEGG_Systemic_Lupus_Erythematosus	-2.17709	-1.8855	59,407
KEGG_Aldosterone_Regulated_Sodium_Reabsorption	-2.17975	-1.88346	59,407
KEGG_Rna_Degradation	-2.18133	-1.8803	59,407
KEGG_Valine_Leucine_And_Isoleucine_Degradation	-2.09389	-1.80695	59,407
KEGG_Nucleotide_Excision_Repair	-2.09389	-1.80695	59,407
KEGG_Pantothenate_And_Coa_Biosynthesis	-2.00949	-1.73619	59,407
KEGG_N_Glycan_Biosynthesis	-2.01295	-1.73515	59,407
KEGG_O_Glycan_Biosynthesis	-2.00181	-1.73296	59,407
KEGG_Renin_Angiotensin_System	-1.93434	-1.66991	59,407
KEGG_Alanine_Aspartate_And_Glutamate_Metabolism	-1.90324	-1.64317	59,407
KEGG_Glutathione_Metabolism	-1.86409	-1.60834	59,407
KEGG_Butanoate_Metabolism	-1.81215	-1.56068	59,407
KEGG_Snare_Interactions_In_Vesicular_Transport	-1.64894	-1.4017	59,407
KEGG_Sphingolipid_Metabolism	-1.61157	-1.36853	59,407
KEGG_Mismatch_Repair	-1.5734	-1.33864	59,407
KEGG_Pyruvate_Metabolism	-1.57543	-1.33655	59,407
KEGG_Aminoacyl_Trna_Biosynthesis	-1.54046	-1.30977	59,407
KEGG_Porphyrin_And_Chlorophyll_Metabolism	-1.54046	-1.30977	59,407

**Fig. 4:**
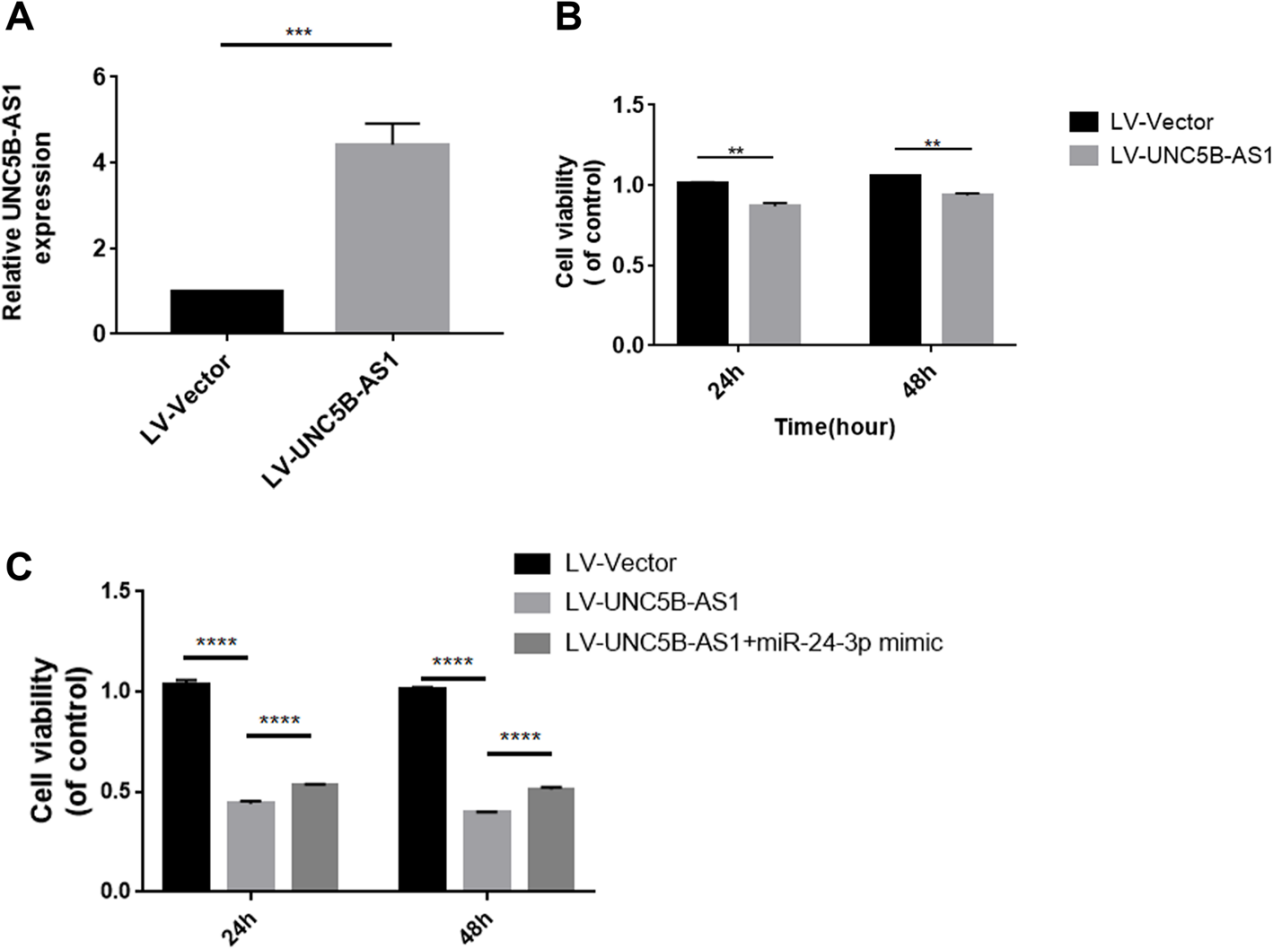
UNC5B-AS1 enhanced the proliferative ability of GBM cells. **A** Efficiency of UNC5B-AS1 overexpression in U87MG cells. **B** UNC5B-AS1 inhibited cell proliferation. Time is represented on the horizontal axis, and cell viability is represented on the vertical axis. The black and grey pillars indicate LV-Vector and LV-UNC5B-AS1, respectively. Unpaired Student’s t test was utilized in a, and two-way ANOVA with Sidak’s multiple comparisons test was utilized in b. **C** Overexpression of miR-24-3p attenuated the effects of UNC5B-AS1 on GBM cell proliferation. Multiple t test was utilized in **B**, Two-way ANOVA with Tukey’s multiple comparisons test was utilized in **C**, **p < 0.01, ***p < 0.001 and ****p < 0.0001

To verify our bioinformatic analysis, we conducted in vitro analysis to explore the molecular biological roles of UNC5B-AS1 in GBM cells. The CCK-8 test was used to assess UNC5B-AS1's capacity for cell proliferation. Our results indicated that in contrast to LV-Vector group, the expression of UNC5B-AS1 in the LV-UNC5B-AS1 group was especially higher (Fig. 4A). The results demonstrated that cell proliferation in the LV-UNC5B-AS1 group was inhibited in contrast to that in the LV-Vector group (Fig. 4B). Overall, based on these findings, the function of UNC5B-AS1 could regulate the proliferative ability of GBM cells by sponging miR-24-3p.

### UNC5B-AS1 regulate cell proliferation through the ceRNA sponging of miR-24-3p

**Fig. 5:**
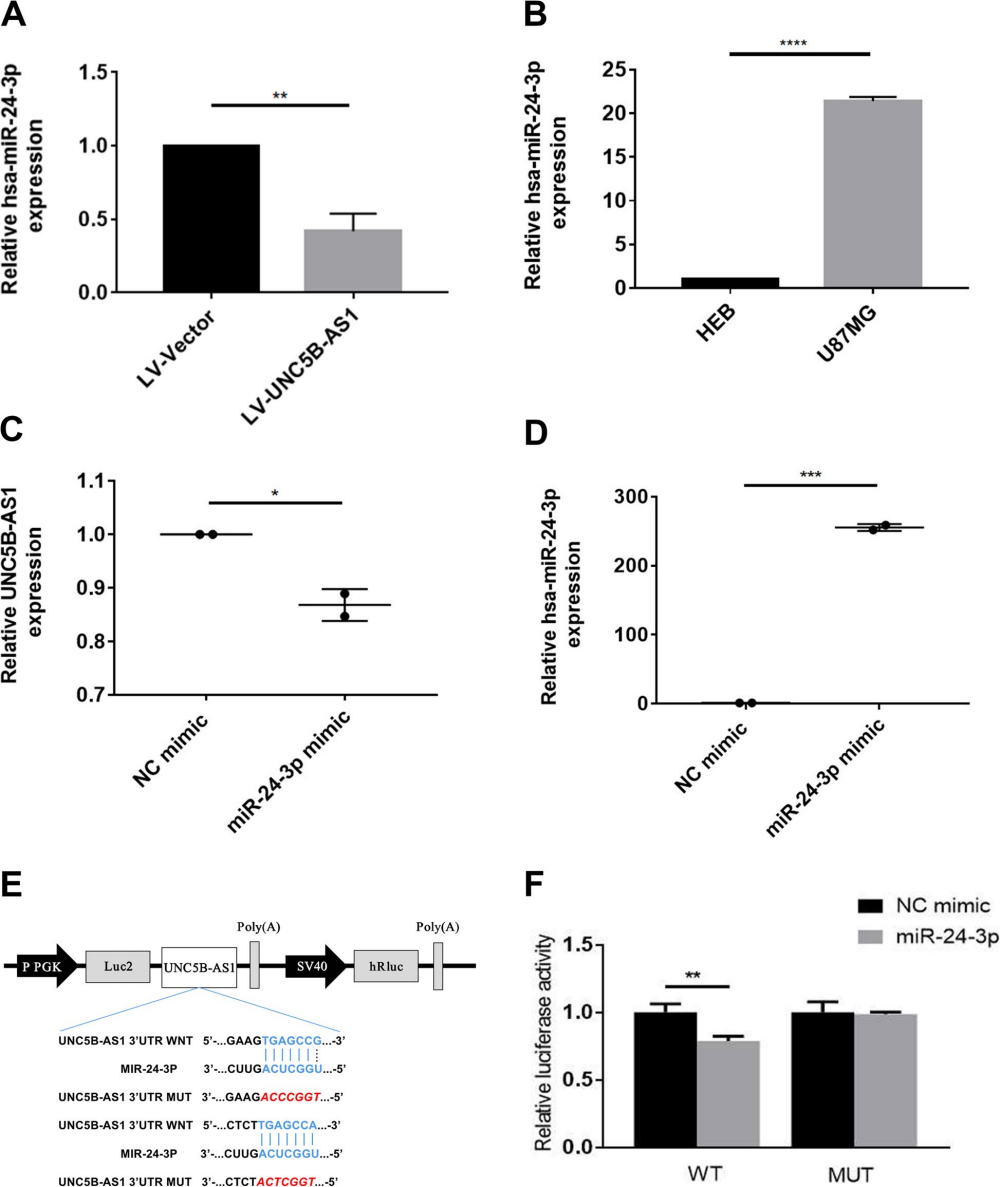
MiR-24-3p is sponged by UNC5B-AS1 in GBM. **A** UNC5B-AS1 overexpression inhibited miR-24-3p expression in the U87MG cell line. **B** The expression of miR-24-3p was significantly high in GBM. **C** Enhanced expression of miR-24-3p suppressed UNC5B-AS1 expression in the U87MG cell line. **D** Efficiency of the miR-24-3p mimics. Unpaired Student’s t test was utilized in **A**-**D**, *p < 0.05, **p < 0.01 and ****p < 0.0001. **E** Possible binding locations between UNC5B-AS1 and miR-24-3p. **F** The relative luciferase activity after transfection of both the miR-24-3p mimic and the 3’UTR of UNC5B-AS1 mRNA into U87MG cells

To detect the potential binding sites of UNC5B-AS1**,** qRT‒PCR analysis was utilized, and the results indicated that increased expression of UNC5B-AS1 repressed the expression of miR-24-3p (Fig. 5A). Figure 5B shows that miR-24-3p expression was significantly higher in U87MG cells than in HEB cells. Moreover, miR-24-3p overexpression inhibited the expression of UNC5B-AS1 in U87MG cells (Fig. 5C). Figure 5D demonstrates the miR-24-3p mimic transfection efficiency.

Additionally, the probable binding regions between UNC5B-AS1 and miR-24-3p were estimated using RNAhybrid (Fig. 5E). The dual luciferase reporter assay revealed that, in comparison to wild-type UNC5B-AS1 mimics of the NC group, wild-type UNC5B-AS1 transfected with miR-24-3p mimics had suppressed luciferase activity, whereas the luciferase activity of the mutant-type UNC5B-AS1 mimic NC group and mutant-type UNC5B-AS1 miR-24-3p mimics were similar (Fig. 5F). These findings predicted that UNC5B-AS1 may target miR-24-3p, thereby affect the biological process of GBM.

We further evaluated the association of UNC5B-AS1 and miR-24-3p in U87MG cells by CCK-8 experiments. The findings revealed that UNC5B-AS1 overexpression suppressed GBM cell proliferation, whereas cotransfection of miR-24-3p mimics and LV-UNC5B-AS1 effectively diminished the inhibitory effects of UNC5B-AS1 overexpression on U87MG cells (Fig. 4C).

## Conclusion

Long noncoding RNAs (lncRNAs), a recent innovation in molecular biology, are crucial for controlling gene expression at the stages of the epigenome, transcription and posttranscription [30]. LncRNAs, which appear to act as miRNA sponges, can negatively attenuate protein-coding gene expression and then regulate cell differentiation and progression in various diseases, including GBM and other cancers. Numerous lncRNAs and their sponged targets have been identified as both diagnostic and therapeutic targets in the development of GBM tumours, such as LINC02015, H19, KIAA0495, AC068888.1 and MALAT1 [31–34]. In spite of the ceRNAs theory has made considerable progress in the development of diagnostic markers, little is known about the role of lncRNAs and its combined targets in GBM development. Moreover, the lack of sensitive and specific GBM biomarkers has resulted in overdiagnosis and overtreatment [35]. Hence, for the effective diagnosis and treatment of GBM, there is an urgent need to discover GBM-related lncRNAs and uncover their potential mechanisms.

UNC5B-AS1 is a novel carcinogenic lncRNA that has been found in thyroid papillary carcinoma, ovarian cancer, colon cancer and prostate cancer. For example, UNC5B-AS1 was considered to exert a crucial role in cell growth and metastasis of papillary thyroid cancer (PTC) and may be a therapeutic target for PTC [36]. In addition, UNC5B-AS1 accelerates the proliferation, migration, and epithelial-mesenchymal transition (EMT) of hepatocellular carcinoma (HCC) cells via sponging miR-4306 [37]. However, its exact biological roles and direct targets in the progression of GBM are still unknown. In this study, we aimed to discover the roles of UNC5B-AS1 in glioma and investigate its potential mechanisms in the development of GBM. Additionally, many therapeutic targets of UNC5B-AS1 are needed to be explored for GBM treatment.

MiRNAs act as post-transcriptional regulators of gene expression, with 22 ~ 25 nucleotides in length. MiRNAs can be significantly overexpressed in many cancer cells and exert biological roles via interacting with target mRNAs. For example, miR-22-3p can target Wnt-signaling pathway-associated mRNAs and promote the carcinogenesis of colon cancer cells [38]. In glioma, miR-24-3p can specially target the 3’ UTR of MAX interactor 1 (Maxi 1) to promote the cell proliferation [39]. Based on the background above, in this study, we performed bioinformatic analysis and survival analysis to screen potential miRNAs of UNC5B-AS1 and explore its biological functions in vitro. We assumed that miR-24-3p might act as a target of UNC5B-AS1 and be involved in regulating tumor development, which will provide a deeper understanding of GBM development.

To validate our assumptions, we aimed to determine abnormally expressed lncRNAs and miRNAs, as well as their biological regulatory roles in GBM cells. In this study, we firstly discovered the roles of UNC5B-AS1 was downregulated in GBM cells, which is consistent with the bioinformatic analysis. In addition, we found that overexpressed UNC5B-AS1 inhibited cell proliferation, indicates that UNC5B-AS1 might act as tumor suppressor in GBM. We also found that miR-24-3p can sponge UNC5B-AS1 to reverses the effect of cell proliferation, demonstrates the potential biological mechanisms of UNC5B-AS1 in GBM. Moreover, miR-24-3p was significantly associated with LGG patients’ prognosis, indicates that UNC5B-AS1 may interact with miR-24-3p to influence patents’ survival. However, more experiments are needed to further test these hypotheses. Based on our results, a novel UNC5B-AS1/miR-24-3p regulation axis was constructed, which may act a key pathway in the GBM development and become a new therapeutic application of GBM in the foreseeable future.

The current work also has a few shortcomings. First, in the TCGA dataset, we solely examined and verified the biological roles of lncRNA biomarkers, and no other database of lncRNA expression data associated with GBM was used for further validation. Second, the interaction between UNC5B-AS1 and miR-24-3p were only explored by the qRT-PCR analysis and luciferase assay. More experiments, including RNA pull down and immunoprecipitation (RIP). However, we will process more intensive research on anther experiments in the future. Finally, this study used a database mining design without validation in fresh samples due to our limited conditions, such as patients’ tissues and animal experimental studies. We hope to perfect our work in the future.

In conclusion, our study suggested the potential functions of the UNC5B-AS1-miR-24-3p regulatory link, which may serve as cutting-edge potential biomarkers for GBM diagnosis and prognosis.

### Supplementary Information

**Supplementary Material 1.**

## Data Availability

These data utilized to back up the study’s findings is supplied in the article.
