# Supplementary material for: Long noncoding RNA UNC5B-AS1 suppresses cell proliferation by sponging miR-24-3p in glioblastoma multiforme
Source: BMC Med Genomics. 2024 Apr 9;17:83. doi: 10.1186/s12920-024-01851-5 (PMC11003007; doi:10.1186/s12920-024-01851-5)
Supplement: Supplementary file 1 — Supplementary Material 1. [file 12920_2024_1851_MOESM1_ESM.zip › Additional file 1/A.Table 1.docx]

**A.Table 1.** Upregulated lncRNAs

| LncRNAs name | logFC | logCPM | PValue | FDR |
| --- | --- | --- | --- | --- |
| RP11-124N14.3 | 5.650400763 | 8.47637469 | 1.48E-17 | 9.35E-16 |
| RP11-347E10.1 | 9.866548995 | 7.628905953 | 7.35E-14 | 2.91E-12 |
| WARS2-IT1 | 5.535365041 | 6.998550029 | 9.67E-14 | 3.81E-12 |
| HOXD-AS2 | 9.578992781 | 7.34625206 | 2.77E-13 | 9.91E-12 |
| FOXD3-AS1 | 7.686804092 | 8.775648791 | 4.36E-13 | 1.52E-11 |
| CRNDE | 4.916857402 | 9.728577083 | 3.85E-12 | 1.22E-10 |
| HOXA-AS3 | 8.878876744 | 6.664140205 | 1.64E-11 | 4.69E-10 |
| RP11-161H23.9 | 4.353392801 | 5.735997399 | 7.57E-11 | 1.99E-09 |
| LINC01579 | 4.880110043 | 10.58065151 | 9.63E-11 | 2.49E-09 |
| RP11-126K1.6 | 2.914960522 | 8.426470789 | 1.05E-10 | 2.68E-09 |
| RP11-3P17.5 | 3.246406273 | 6.335085342 | 1.21E-10 | 3.04E-09 |
| RP11-428J1.5 | 2.75926365 | 6.967073995 | 1.52E-10 | 3.80E-09 |
| HAS2-AS1 | 3.043596892 | 7.937737754 | 2.06E-10 | 5.12E-09 |
| RP11-257P3.3 | 3.51394946 | 5.110590061 | 2.17E-10 | 5.37E-09 |
| HOTAIRM1 | 5.971001146 | 9.396547227 | 3.96E-10 | 9.42E-09 |
| CTD-2510F5.4 | 2.64405579 | 7.395452477 | 4.06E-10 | 9.63E-09 |
| RP11-705C15.3 | 2.370822899 | 7.437361847 | 7.48E-10 | 1.70E-08 |
| CTD-2006C1.2 | 2.03151542 | 8.150072802 | 8.30E-10 | 1.87E-08 |
| HOXA-AS2 | 8.628850918 | 8.335959709 | 1.32E-09 | 2.88E-08 |
| RP11-671J11.5 | 5.585614513 | 3.75066844 | 1.78E-09 | 3.84E-08 |
| RP11-299L17.3 | 6.304035775 | 6.941734099 | 1.81E-09 | 3.87E-08 |
| RP11-452L6.6 | 2.747001129 | 6.701336981 | 1.91E-09 | 4.07E-08 |
| RP11-698N11.2 | 6.134617619 | 8.758098696 | 2.66E-09 | 5.55E-08 |
| RP11-661A12.8 | 2.008119013 | 6.874211486 | 3.41E-09 | 6.89E-08 |
| WEE2-AS1 | 2.472942135 | 8.943612951 | 4.05E-09 | 8.08E-08 |
| RP11-420L9.5 | 2.774102482 | 9.790026771 | 4.12E-09 | 8.19E-08 |
| TBX2-AS1 | 3.270598713 | 8.205320527 | 5.63E-09 | 1.09E-07 |
| RP11-403A3.3 | 5.055766886 | 11.9878461 | 6.69E-09 | 1.28E-07 |
| ARHGAP31-AS1 | 2.75548112 | 5.641856093 | 1.11E-08 | 2.08E-07 |
| AC002456.2 | 3.586567795 | 7.753117288 | 2.30E-08 | 4.02E-07 |
| CTD-3035K23.7 | 3.284572638 | 7.735502647 | 3.61E-08 | 6.15E-07 |
| PVT1 | 2.445869567 | 9.243592914 | 3.69E-08 | 6.26E-07 |
| HOXC-AS2 | 8.408375655 | 6.210491287 | 3.79E-08 | 6.41E-07 |
| CTB-113P19.1 | 3.28669061 | 4.370992252 | 4.61E-08 | 7.68E-07 |
| LBX2-AS1 | 3.385013608 | 8.055425903 | 5.60E-08 | 9.21E-07 |
| LINC01587 | 6.406649467 | 5.552322497 | 6.37E-08 | 1.04E-06 |
| RP11-175K6.2 | 3.133332003 | 6.29628216 | 6.59E-08 | 1.06E-06 |
| UBE2E1-AS1 | 2.821410888 | 6.166318774 | 8.51E-08 | 1.34E-06 |
| PSMB8-AS1 | 2.561955663 | 9.925049532 | 8.56E-08 | 1.34E-06 |
| AC092675.3 | 3.631578251 | 7.053841195 | 9.14E-08 | 1.42E-06 |
| TFAP2A-AS1 | 5.138470076 | 7.199906882 | 9.15E-08 | 1.42E-06 |
| DARS-AS1 | 2.320405983 | 6.352565438 | 9.39E-08 | 1.45E-06 |
| RP11-356I2.4 | 2.988642413 | 6.727647823 | 9.99E-08 | 1.53E-06 |
| CTD-2270L9.4 | 2.196214002 | 7.380812852 | 1.05E-07 | 1.60E-06 |
| RP11-403A3.2 | 4.426446284 | 8.157211817 | 1.24E-07 | 1.86E-06 |
| RP11-204L24.2 | 2.924895884 | 5.7990603 | 1.39E-07 | 2.07E-06 |
| RP11-932O9.10 | 3.947438048 | 4.407784869 | 1.44E-07 | 2.13E-06 |
| RP11-19E11.1 | 6.398340016 | 6.172530007 | 1.48E-07 | 2.18E-06 |
| LINC00511 | 2.68317865 | 12.36523557 | 1.54E-07 | 2.26E-06 |
| AC010729.1 | 3.446437022 | 5.879568827 | 1.62E-07 | 2.37E-06 |
| RP11-452F19.3 | 2.316391313 | 8.665745671 | 1.69E-07 | 2.46E-06 |
| CTC-498J12.1 | 3.754670549 | 5.484911488 | 1.76E-07 | 2.55E-06 |
| CTD-2116N20.1 | 4.913609535 | 4.253821629 | 1.82E-07 | 2.62E-06 |
| RP11-834C11.4 | 2.92893712 | 10.28477621 | 2.02E-07 | 2.87E-06 |
| HCG15 | 2.680626034 | 6.163645201 | 2.18E-07 | 3.07E-06 |
| GNAS-AS1 | 2.826082474 | 6.117746621 | 2.33E-07 | 3.27E-06 |
| NFIA-AS2 | 3.878374487 | 9.740246352 | 2.83E-07 | 3.94E-06 |
| RP11-434E6.4 | 5.663795234 | 4.88398769 | 2.86E-07 | 3.96E-06 |
| CARD8-AS1 | 2.458998308 | 8.740588668 | 2.90E-07 | 4.01E-06 |
| HCG16 | 3.064859081 | 4.855461779 | 3.36E-07 | 4.59E-06 |
| AC006126.4 | 4.611127862 | 5.326797139 | 3.61E-07 | 4.89E-06 |
| LINC01451 | 4.290160295 | 5.019472956 | 3.65E-07 | 4.93E-06 |
| RP11-10A14.4 | 5.669538433 | 3.792065969 | 3.85E-07 | 5.18E-06 |
| RP3-510D11.2 | 3.945001567 | 6.671156305 | 4.05E-07 | 5.41E-06 |
| LINC00704 | 5.126207166 | 4.410487906 | 4.31E-07 | 5.73E-06 |
| LEF1-AS1 | 3.938910807 | 6.876892862 | 4.46E-07 | 5.91E-06 |
| LINC01571 | 6.127092351 | 5.291870123 | 4.50E-07 | 5.95E-06 |
| DLGAP1-AS2 | 2.021098125 | 8.707970952 | 5.40E-07 | 7.07E-06 |
| RP11-84A19.3 | 5.706105345 | 8.195766104 | 5.40E-07 | 7.07E-06 |
| LINC01426 | 4.137954696 | 7.868954061 | 5.41E-07 | 7.07E-06 |
| HOTAIR | 9.032828993 | 8.142454014 | 5.95E-07 | 7.73E-06 |
| LINC01358 | 3.554357391 | 6.037174685 | 6.30E-07 | 8.12E-06 |
| RP11-742B18.1 | 7.694602574 | 6.816335812 | 6.40E-07 | 8.23E-06 |
| RP11-435O5.2 | 3.839905804 | 5.206554452 | 8.05E-07 | 1.01E-05 |
| RP11-713M15.2 | 2.657905522 | 5.927066151 | 8.10E-07 | 1.02E-05 |
| HOXC-AS1 | 8.472686128 | 6.274033387 | 8.92E-07 | 1.11E-05 |
| RP11-220C2.1 | 5.881459082 | 3.956632478 | 9.12E-07 | 1.13E-05 |
| RP11-756G20.1 | 2.956130868 | 5.717065801 | 9.60E-07 | 1.18E-05 |
| RP11-379B8.1 | 3.5441383 | 5.323182764 | 9.80E-07 | 1.20E-05 |
| AC011899.9 | 3.425145367 | 8.095745408 | 1.00E-06 | 1.22E-05 |
| RP11-142A22.4 | 4.408785682 | 5.082975093 | 1.01E-06 | 1.23E-05 |
| CTB-193M12.5 | 2.009537252 | 9.355407486 | 1.07E-06 | 1.29E-05 |
| LINC01550 | 2.302722341 | 7.980693345 | 1.21E-06 | 1.44E-05 |
| WNT5A-AS1 | 3.283175554 | 5.497747024 | 1.31E-06 | 1.57E-05 |
| CTB-70G10.1 | 6.18803366 | 4.200424517 | 1.32E-06 | 1.57E-05 |
| PIK3CD-AS2 | 4.07941488 | 7.256197468 | 1.43E-06 | 1.70E-05 |
| TMPO-AS1 | 2.098358523 | 7.968486224 | 1.58E-06 | 1.86E-05 |
| FOXP4-AS1 | 3.144570431 | 4.840098699 | 1.58E-06 | 1.86E-05 |
| HOXB-AS1 | 5.405847316 | 7.158586761 | 1.77E-06 | 2.07E-05 |
| RP11-524D16__A.3 | 4.733358159 | 8.146093611 | 1.81E-06 | 2.11E-05 |
| CSTF3-AS1 | 2.659728183 | 5.034258427 | 1.82E-06 | 2.12E-05 |
| AC021188.4 | 2.582895503 | 6.845537441 | 1.83E-06 | 2.12E-05 |
| AC005083.1 | 3.937981993 | 5.335899337 | 1.83E-06 | 2.12E-05 |
| LINC01152 | 2.1309039 | 9.344542803 | 1.85E-06 | 2.13E-05 |
| CTD-2369P2.4 | 3.800060626 | 3.391879046 | 1.85E-06 | 2.13E-05 |
| RP11-134N1.2 | 4.478102646 | 5.387460101 | 1.96E-06 | 2.24E-05 |
| HOXC-AS3 | 6.889100043 | 4.808686052 | 2.18E-06 | 2.49E-05 |
| RP11-148K1.12 | 2.130816984 | 6.845426698 | 2.30E-06 | 2.62E-05 |
| RP4-580N22.2 | 2.618717871 | 4.814726741 | 2.37E-06 | 2.68E-05 |
| RP11-219B4.5 | 3.897250387 | 3.478431019 | 2.39E-06 | 2.70E-05 |
| RP1-65J11.1 | 3.228646645 | 7.90752167 | 2.45E-06 | 2.76E-05 |
| RP11-298I3.4 | 2.458745406 | 5.746476162 | 2.51E-06 | 2.82E-05 |
| RP11-219G17.4 | 5.394271422 | 6.018314618 | 2.56E-06 | 2.86E-05 |
| RP11-157F20.3 | 3.588798209 | 4.425787453 | 2.77E-06 | 3.08E-05 |
| CTC-548K16.2 | 3.175578794 | 4.482806641 | 2.82E-06 | 3.12E-05 |
| ZNF503-AS1 | 2.427138743 | 5.052487263 | 2.85E-06 | 3.15E-05 |
| HOXA11-AS | 7.192506002 | 6.361870582 | 2.86E-06 | 3.15E-05 |
| ASAP1-IT2 | 3.285569365 | 5.16763702 | 3.12E-06 | 3.39E-05 |
| RP11-503C24.2 | 7.021138276 | 4.924546193 | 3.42E-06 | 3.67E-05 |
| RP11-360N9.2 | 2.874002228 | 4.810637715 | 3.43E-06 | 3.67E-05 |
| CTD-2227E11.1 | 4.535351795 | 4.903394888 | 3.76E-06 | 3.97E-05 |
| RP11-367G18.1 | 3.714382818 | 4.229206818 | 3.83E-06 | 4.03E-05 |
| RP11-76E17.3 | 5.18606712 | 5.767699515 | 3.89E-06 | 4.09E-05 |
| FOXD2-AS1 | 3.532923912 | 7.954461032 | 4.43E-06 | 4.63E-05 |
| RP3-460G2.2 | 5.735994336 | 8.079842507 | 4.57E-06 | 4.75E-05 |
| RP11-230B22.1 | 2.222398981 | 6.78434037 | 5.11E-06 | 5.29E-05 |
| RP11-259N19.1 | 2.13490234 | 8.240296773 | 5.17E-06 | 5.33E-05 |
| RP11-142C4.6 | 3.480496601 | 5.05759774 | 5.19E-06 | 5.34E-05 |
| AC147651.4 | 3.123496719 | 7.719211429 | 5.20E-06 | 5.34E-05 |
| RP11-565P22.2 | 5.503302445 | 6.676976928 | 5.28E-06 | 5.41E-05 |
| RP11-184M15.1 | 5.492844589 | 4.749017047 | 5.28E-06 | 5.41E-05 |
| IQCA1-AS1 | 4.448394241 | 4.449901693 | 5.42E-06 | 5.52E-05 |
| LINC01349 | 5.37105777 | 3.567098342 | 5.54E-06 | 5.63E-05 |
| AJ011932.1 | 3.398120462 | 5.398703532 | 5.61E-06 | 5.70E-05 |
| RP11-437L7.1 | 6.017754529 | 5.870608087 | 5.74E-06 | 5.82E-05 |
| FIRRE | 2.244148097 | 7.075358669 | 5.85E-06 | 5.91E-05 |
| MED4-AS1 | 2.116648622 | 4.179651728 | 5.99E-06 | 6.03E-05 |
| RP11-54A9.1 | 3.982584297 | 5.861152587 | 6.17E-06 | 6.20E-05 |
| ATP2A1-AS1 | 2.226600088 | 6.982660851 | 6.87E-06 | 6.86E-05 |
| LINC01235 | 3.5912682 | 8.763318986 | 7.11E-06 | 7.05E-05 |
| AC009005.2 | 2.720924339 | 7.222363718 | 7.65E-06 | 7.54E-05 |
| RP11-2K6.1 | 2.288961825 | 4.426047859 | 7.68E-06 | 7.57E-05 |
| LINC00519 | 3.735042047 | 5.138181124 | 7.75E-06 | 7.63E-05 |
| RP11-696D21.2 | 4.329029836 | 6.484916191 | 8.40E-06 | 8.21E-05 |
| NOVA1-AS1 | 4.080015764 | 7.550650849 | 8.46E-06 | 8.25E-05 |
| AC010524.4 | 5.069782591 | 3.356494778 | 8.77E-06 | 8.52E-05 |
| CTD-2023M8.1 | 3.620684455 | 4.162982364 | 9.17E-06 | 8.82E-05 |
| C17orf82 | 2.923336793 | 5.963114274 | 9.30E-06 | 8.89E-05 |
| RP11-158M2.3 | 2.336883259 | 8.114577175 | 9.94E-06 | 9.45E-05 |
| LINC01143 | 5.601793052 | 3.738453593 | 1.04E-05 | 9.86E-05 |
| RP11-218E20.3 | 2.731570317 | 7.324063809 | 1.05E-05 | 9.87E-05 |
| AC021224.1 | 2.032823103 | 5.124864323 | 1.05E-05 | 9.89E-05 |
| RP11-462G2.1 | 4.553146235 | 3.048879958 | 1.09E-05 | 0.000102748 |
| MEOX2-AS1 | 5.808622887 | 9.168725219 | 1.16E-05 | 0.00010858 |
| RP11-80A15.1 | 2.523822045 | 4.817573631 | 1.21E-05 | 0.000112501 |
| LINC01342 | 5.963698683 | 4.022233565 | 1.24E-05 | 0.000114495 |
| LINC01198 | 6.855836168 | 7.664546813 | 1.34E-05 | 0.00012231 |
| RP11-141O11.2 | 3.038522233 | 5.127367088 | 1.35E-05 | 0.000123229 |
| RP11-554E23.4 | 2.486648826 | 3.749726468 | 1.47E-05 | 0.000132633 |
| AC009505.2 | 3.066076703 | 3.375637655 | 1.58E-05 | 0.000141853 |
| WI2-87327B8.2 | 5.146539448 | 5.507723828 | 1.69E-05 | 0.000150549 |
| RP11-1334A24.5 | 3.273986632 | 6.155266365 | 1.72E-05 | 0.000152573 |
| RP11-48F14.1 | 5.668600038 | 3.791336691 | 1.75E-05 | 0.000154749 |
| LINC00466 | 5.595385145 | 3.735390344 | 1.81E-05 | 0.000159272 |
| RP11-589C21.6 | 3.326523695 | 3.543339151 | 1.81E-05 | 0.000159515 |
| RP11-4C20.3 | 2.198515999 | 5.237944181 | 1.82E-05 | 0.00015978 |
| LINC01385 | 6.199135548 | 4.20435493 | 1.83E-05 | 0.000160311 |
| RP11-647K16.1 | 2.168581078 | 4.77417026 | 1.86E-05 | 0.000162527 |
| MIR155HG | 2.916281646 | 7.57955844 | 1.99E-05 | 0.000173811 |
| RP11-670E13.6 | 2.716273381 | 5.29941911 | 2.00E-05 | 0.000174484 |
| RP11-473M20.16 | 3.22786696 | 4.972044917 | 2.03E-05 | 0.000177101 |
| RP3-337O18.9 | 3.126616379 | 5.407566776 | 2.23E-05 | 0.000192119 |
| AC133644.2 | 3.321126841 | 5.46881575 | 2.28E-05 | 0.000196097 |
| RP11-329L6.2 | 2.978833522 | 6.526132742 | 2.29E-05 | 0.00019677 |
| LINC01158 | 2.462927368 | 11.80140177 | 2.31E-05 | 0.000198384 |
| RP11-218E20.5 | 3.020646883 | 3.927228203 | 2.43E-05 | 0.000207271 |
| CTD-2263F21.1 | 4.123488522 | 4.566827777 | 2.51E-05 | 0.000212735 |
| CMB9-22P13.1 | 2.097127186 | 8.633169435 | 2.72E-05 | 0.000229964 |
| RP11-120D5.1 | 2.113930602 | 4.841959047 | 2.76E-05 | 0.00023226 |
| RP11-222K16.2 | 3.505153017 | 4.735427208 | 2.78E-05 | 0.000233576 |
| CTC-255N20.1 | 2.379376008 | 5.974643365 | 2.81E-05 | 0.000236272 |
| RP11-879F14.2 | 2.611455936 | 5.852607418 | 2.88E-05 | 0.000240368 |
| RP1-232P20.1 | 4.794526945 | 5.194248957 | 2.94E-05 | 0.000245347 |
| AC069513.4 | 2.697954785 | 5.702658567 | 2.95E-05 | 0.000245661 |
| LINC01127 | 3.295674372 | 6.420249715 | 3.24E-05 | 0.000267016 |
| LINC01504 | 2.637905389 | 4.812129042 | 3.25E-05 | 0.000267474 |
| RP11-806H10.4 | 3.453391236 | 6.816117822 | 3.32E-05 | 0.000273208 |
| ACTN1-AS1 | 3.633883617 | 3.795726403 | 3.44E-05 | 0.000282768 |
| CTC-548K16.1 | 3.649954508 | 5.946324694 | 3.55E-05 | 0.000290468 |
| AP000280.66 | 4.239739069 | 4.685512002 | 3.61E-05 | 0.000294147 |
| LINC01132 | 2.134544671 | 6.067176267 | 3.70E-05 | 0.000299903 |
| RP3-369A17.4 | 2.684097883 | 5.676720822 | 3.95E-05 | 0.000318145 |
| CTD-2313J17.5 | 2.068210875 | 4.745995301 | 3.97E-05 | 0.000319283 |
| RP1-79C4.4 | 2.989490168 | 6.799455253 | 4.10E-05 | 0.000329779 |
| HOXC13-AS | 8.443839404 | 6.243123985 | 4.13E-05 | 0.00033103 |
| LAMA5-AS1 | 2.891772507 | 6.265378411 | 4.21E-05 | 0.00033643 |
| RP11-734K23.9 | 2.733822386 | 3.448738261 | 4.53E-05 | 0.00035932 |
| RP11-1136G4.2 | 4.555417482 | 5.965518102 | 4.56E-05 | 0.000361339 |
| RP11-2B6.3 | 2.09471224 | 5.120490831 | 4.69E-05 | 0.000368754 |
| RP11-872D17.4 | 2.208664965 | 4.123173771 | 4.69E-05 | 0.000368754 |
| LINC00475 | 5.196163649 | 7.815476943 | 4.70E-05 | 0.000368754 |
| RP3-439F8.1 | 3.92938249 | 5.792664396 | 5.20E-05 | 0.000403864 |
| RP11-1112J20.2 | 2.115053024 | 3.949556232 | 5.30E-05 | 0.000410578 |
| RP11-472K17.3 | 2.61110798 | 6.565869326 | 5.31E-05 | 0.000411295 |
| RP11-93K22.13 | 3.282269062 | 3.49832958 | 5.34E-05 | 0.000412822 |
| CTB-96E2.6 | 2.508560828 | 4.976795197 | 5.54E-05 | 0.000425719 |
| H19 | 7.483571273 | 11.97349211 | 5.55E-05 | 0.000425904 |
| CH507-528H12.1 | 6.91862687 | 4.771382625 | 5.61E-05 | 0.000429879 |
| AC002044.4 | 4.594970323 | 3.048346162 | 5.87E-05 | 0.000448168 |
| RP11-497H16.9 | 4.481751311 | 3.909583972 | 5.96E-05 | 0.000454364 |
| RP11-175K6.1 | 2.364364471 | 5.831309719 | 5.97E-05 | 0.00045481 |
| RP3-431P23.5 | 5.214261658 | 6.991497922 | 6.04E-05 | 0.0004596 |
| PCED1B-AS1 | 2.493491189 | 10.20550334 | 6.25E-05 | 0.00047299 |
| RP11-139K4.2 | 3.262321411 | 4.120864156 | 6.30E-05 | 0.000476163 |
| RP11-28H5.2 | 2.324486584 | 4.190837214 | 6.36E-05 | 0.000480061 |
| RP11-122C5.3 | 3.411153784 | 5.313810489 | 6.59E-05 | 0.000496115 |
| LLNLR-304G9.1 | 2.896299807 | 4.57483516 | 6.65E-05 | 0.000499667 |
| XXbac-BPG157A10.21 | 2.092542906 | 5.114504174 | 6.79E-05 | 0.000508528 |
| MNX1-AS1 | 7.128798648 | 5.02430483 | 6.82E-05 | 0.000509814 |
| RP11-834C11.11 | 2.836291919 | 4.875638318 | 6.84E-05 | 0.000510795 |
| CTB-41I6.2 | 2.953613141 | 5.165683308 | 6.92E-05 | 0.000513177 |
| CTD-2521M24.9 | 2.833449979 | 9.497012698 | 6.99E-05 | 0.000516765 |
| KB-1440D3.13 | 2.480722287 | 4.862588819 | 6.99E-05 | 0.000516765 |
| LINC01366 | 2.909860598 | 4.234226596 | 7.05E-05 | 0.000519495 |
| RP4-607I7.1 | 2.953280961 | 5.978727983 | 7.09E-05 | 0.000521619 |
| RP11-145H9.3 | 3.337746305 | 3.558287881 | 7.13E-05 | 0.000524044 |
| RP11-116D17.3 | 5.609452259 | 3.7516337 | 7.23E-05 | 0.000529997 |
| LINC01506 | 3.480113169 | 4.758345856 | 7.30E-05 | 0.000533387 |
| LINC00908 | 3.771866396 | 6.376866197 | 7.46E-05 | 0.0005425 |
| TMEM92-AS1 | 2.693718744 | 4.56866535 | 7.58E-05 | 0.000550569 |
| RP11-752L20.3 | 2.420732975 | 7.224004949 | 8.03E-05 | 0.000578404 |
| AC008278.2 | 4.522371461 | 2.999344955 | 8.13E-05 | 0.000584783 |
| RP11-835E18.5 | 2.08923233 | 3.922053152 | 8.19E-05 | 0.000586991 |
| RP11-53B2.6 | 2.033171283 | 4.864285894 | 8.46E-05 | 0.000605103 |
| RP11-244M2.1 | 3.512649214 | 7.94313445 | 8.71E-05 | 0.000619936 |
| LINC01096 | 5.826508356 | 3.914295462 | 8.75E-05 | 0.000621691 |
| RP11-286H14.8 | 3.007635089 | 5.282906106 | 8.85E-05 | 0.00062787 |
| RP11-86H7.6 | 2.743697194 | 5.601739509 | 8.97E-05 | 0.00063519 |
| RP11-57A1.1 | 2.562770876 | 3.809936451 | 9.00E-05 | 0.00063675 |
| AC010987.6 | 5.17477545 | 3.421015178 | 9.12E-05 | 0.000642923 |
| LINC01206 | 4.996145484 | 7.689743887 | 9.15E-05 | 0.000644065 |
| HAND2-AS1 | 6.380026185 | 6.154140798 | 9.46E-05 | 0.000663404 |
| RP11-477H21.2 | 2.09469547 | 4.891947309 | 9.49E-05 | 0.000664384 |
| RP4-539M6.22 | 3.450504898 | 3.657379771 | 9.78E-05 | 0.000682526 |
| CASC8 | 4.303139263 | 4.29084441 | 0.000101199 | 0.000705705 |
| RP11-10A14.5 | 4.900834764 | 3.234807984 | 0.000103748 | 0.00072268 |
| RP11-1084E5.1 | 4.581206045 | 3.967358598 | 0.000108155 | 0.000749238 |
| RP11-148B18.3 | 4.051707947 | 3.557284794 | 0.000108865 | 0.000752506 |
| RP11-44N12.5 | 4.941840913 | 3.271026119 | 0.000110457 | 0.000762673 |
| RP4-806M20.4 | 2.677509786 | 4.237944995 | 0.000111954 | 0.000771318 |
| RP11-448P19.1 | 3.513762937 | 6.014430059 | 0.000114018 | 0.00078212 |
| RP11-315A16.1 | 4.241829023 | 5.497055708 | 0.000116654 | 0.00079501 |
| RP11-8L8.2 | 2.904700216 | 6.000452973 | 0.000117037 | 0.000796745 |
| RP11-162J8.3 | 3.016085166 | 4.531344476 | 0.000117161 | 0.000796745 |
| RP11-435O5.7 | 3.638518179 | 3.293183673 | 0.000120569 | 0.000819035 |
| AC064875.2 | 3.200487653 | 9.427305829 | 0.00012133 | 0.000823317 |
| RP11-264B14.2 | 2.970310284 | 3.303901262 | 0.000124857 | 0.000844528 |
| RP11-414H17.5 | 2.338831307 | 4.465971907 | 0.000131288 | 0.000879534 |
| AC002044.1 | 2.928158445 | 3.588146832 | 0.000135204 | 0.000904805 |
| CTD-2035E11.4 | 3.00120729 | 6.557326581 | 0.000146606 | 0.00097182 |
| AP000476.1 | 2.684535818 | 5.238336268 | 0.000149604 | 0.000989619 |
| LINC01150 | 2.316871598 | 5.88328336 | 0.000150867 | 0.000996923 |
