# Supplementary material for: Long noncoding RNA UNC5B-AS1 suppresses cell proliferation by sponging miR-24-3p in glioblastoma multiforme
Source: BMC Med Genomics. 2024 Apr 9;17:83. doi: 10.1186/s12920-024-01851-5 (PMC11003007; doi:10.1186/s12920-024-01851-5)
Supplement: Supplementary file 1 — Supplementary Material 1. [file 12920_2024_1851_MOESM1_ESM.zip › Additional file 1/A.Table 2.docx]

**A.Table 2.** Downregulated lncRNAs

| LncRNAs name | logFC | logCPM | PValue | FDR |
| --- | --- | --- | --- | --- |
| LINC00087 | -4.033015107 | 8.993761905 | 1.92E-48 | 1.21E-44 |
| SNAI3-AS1 | -2.461094672 | 8.285319557 | 1.44E-39 | 3.54E-36 |
| AC011995.1 | -5.860260354 | 3.992825471 | 1.68E-39 | 3.54E-36 |
| RP4-561L24.3 | -2.656005917 | 6.484461151 | 7.41E-39 | 1.17E-35 |
| PWAR6 | -3.414409499 | 10.73425317 | 7.23E-37 | 9.11E-34 |
| RP11-434B12.1 | -3.562019945 | 7.35354798 | 1.73E-36 | 1.82E-33 |
| RP13-514E23.1 | -3.387237061 | 7.876627792 | 7.68E-34 | 6.91E-31 |
| RP11-863P13.4 | -4.889317105 | 5.907128515 | 2.25E-33 | 1.77E-30 |
| RP11-701H24.3 | -4.085314154 | 5.773842571 | 5.25E-32 | 3.68E-29 |
| LLNLR-307A6.1 | -3.760522557 | 4.313771348 | 6.13E-32 | 3.86E-29 |
| CTD-2562J17.6 | -3.521154282 | 10.34726015 | 1.59E-31 | 9.10E-29 |
| RP11-361L15.5 | -4.166760193 | 5.654848725 | 2.01E-30 | 1.05E-27 |
| RP11-215H22.1 | -5.547896974 | 4.751115772 | 3.16E-30 | 1.53E-27 |
| SLC26A4-AS1 | -4.977280426 | 8.273497236 | 3.55E-30 | 1.60E-27 |
| RP11-145P16.2 | -4.140847458 | 4.266876372 | 5.48E-30 | 2.30E-27 |
| PART1 | -3.939442156 | 8.273493062 | 2.05E-29 | 8.07E-27 |
| RP11-81K13.1 | -5.491155697 | 3.702582814 | 1.94E-28 | 7.20E-26 |
| RFPL1S | -4.372191788 | 8.931143842 | 6.48E-28 | 2.27E-25 |
| TINCR | -3.301335802 | 7.720248671 | 2.52E-27 | 7.95E-25 |
| LINC00282 | -4.331870774 | 5.969833701 | 4.80E-27 | 1.44E-24 |
| LINC01007 | -6.755735123 | 5.157188811 | 9.15E-27 | 2.62E-24 |
| AC096772.6 | -2.204266353 | 8.879664893 | 1.23E-26 | 3.37E-24 |
| RP11-13K12.5 | -4.36280956 | 5.980143804 | 1.82E-26 | 4.77E-24 |
| RP3-406A7.7 | -3.411339546 | 6.137960648 | 3.20E-26 | 8.06E-24 |
| RP11-210K20.5 | -3.579015512 | 3.537764506 | 1.58E-25 | 3.82E-23 |
| LINC00086 | -3.060643549 | 10.30619203 | 1.69E-25 | 3.94E-23 |
| RP11-389G6.3 | -5.255109784 | 5.610164998 | 3.34E-25 | 7.53E-23 |
| RP11-497E19.1 | -4.367708061 | 4.970443286 | 3.74E-25 | 8.13E-23 |
| AC061961.2 | -5.381222484 | 3.911019891 | 9.36E-25 | 1.97E-22 |
| RP11-236J17.6 | -5.119392928 | 3.629464131 | 9.76E-25 | 1.98E-22 |
| LINC00641 | -2.758750894 | 10.97230018 | 1.66E-24 | 3.28E-22 |
| RP11-294N21.3 | -3.570396008 | 4.003759782 | 3.20E-24 | 6.12E-22 |
| RP5-907C10.3 | -3.802433466 | 7.851240433 | 5.09E-24 | 9.43E-22 |
| RP11-863P13.6 | -4.958044169 | 3.1792317 | 5.48E-24 | 9.79E-22 |
| SHANK3 | -2.682518036 | 11.41302337 | 5.59E-24 | 9.79E-22 |
| RP11-57H14.4 | -2.236681517 | 9.080773561 | 7.98E-24 | 1.36E-21 |
| KB-1460A1.5 | -2.510039945 | 8.651338811 | 9.42E-24 | 1.56E-21 |
| RP11-139H14.5 | -3.224466494 | 6.982579117 | 1.16E-23 | 1.87E-21 |
| ACAP2-IT1 | -3.044798218 | 4.615660084 | 2.63E-23 | 4.12E-21 |
| AC062028.1 | -4.057744229 | 5.312643346 | 2.68E-23 | 4.12E-21 |
| RP5-1142A6.2 | -2.866123378 | 5.595577111 | 5.31E-23 | 7.96E-21 |
| CTD-2561B21.10 | -3.618517108 | 3.462852093 | 1.02E-22 | 1.49E-20 |
| VIPR1-AS1 | -3.478914156 | 3.742666656 | 1.20E-22 | 1.72E-20 |
| RP11-690D19.3 | -2.400496936 | 9.175862145 | 1.32E-22 | 1.86E-20 |
| CTD-2555A7.2 | -3.95795886 | 5.117178181 | 1.55E-22 | 2.12E-20 |
| INHBA-AS1 | -4.157710037 | 5.225166909 | 1.63E-22 | 2.18E-20 |
| LANCL1-AS1 | -2.70464802 | 5.164607239 | 2.33E-22 | 3.06E-20 |
| RP1-34H18.1 | -4.010800399 | 3.95592878 | 2.44E-22 | 3.13E-20 |
| RP11-87E22.2 | -4.943548469 | 4.207371052 | 2.60E-22 | 3.28E-20 |
| RP11-461L13.3 | -3.268065495 | 3.35748072 | 2.65E-22 | 3.28E-20 |
| AC137723.5 | -3.47861601 | 3.432628809 | 3.82E-22 | 4.63E-20 |
| DCTN1-AS1 | -4.160095689 | 5.678034502 | 5.12E-22 | 6.09E-20 |
| RP11-384L8.1 | -3.377842032 | 5.233909455 | 6.66E-22 | 7.77E-20 |
| LINC00940 | -4.367810139 | 4.968391562 | 7.54E-22 | 8.65E-20 |
| IBA57-AS1 | -2.813865881 | 5.124069289 | 1.38E-21 | 1.56E-19 |
| LINC01140 | -2.59849338 | 5.916286727 | 2.02E-21 | 2.23E-19 |
| AC099684.1 | -5.176141744 | 4.453089785 | 2.13E-21 | 2.31E-19 |
| LINC01106 | -4.594458289 | 4.851538681 | 2.70E-21 | 2.88E-19 |
| U62631.5 | -4.554056429 | 3.58398929 | 3.74E-21 | 3.93E-19 |
| LY86-AS1 | -4.602375282 | 6.536489535 | 4.42E-21 | 4.57E-19 |
| RP11-13K12.1 | -3.789812362 | 5.643727987 | 5.15E-21 | 5.23E-19 |
| CTD-2049O4.1 | -3.358766364 | 5.92498224 | 5.72E-21 | 5.69E-19 |
| RP11-333B11.1 | -5.313847609 | 4.454347333 | 5.78E-21 | 5.69E-19 |
| CTD-2561B21.11 | -2.75560638 | 5.158966173 | 5.88E-21 | 5.70E-19 |
| RP11-507K2.6 | -2.716142609 | 5.831554338 | 9.10E-21 | 8.69E-19 |
| MGAT3-AS1 | -3.665076696 | 3.1016019 | 2.60E-20 | 2.43E-18 |
| LINC00950 | -2.529650979 | 8.991666255 | 2.63E-20 | 2.43E-18 |
| RP11-416I2.1 | -5.170360643 | 6.250085435 | 2.66E-20 | 2.43E-18 |
| RP11-320H14.1 | -4.244722483 | 7.249099886 | 3.13E-20 | 2.82E-18 |
| RP11-958J22.1 | -5.135538983 | 3.54065986 | 3.37E-20 | 3.00E-18 |
| RP11-1299A16.3 | -2.538264405 | 7.097053261 | 5.09E-20 | 4.40E-18 |
| DGCR9 | -3.024265922 | 7.349957437 | 5.10E-20 | 4.40E-18 |
| RP11-574K11.5 | -2.489431591 | 5.194665385 | 1.17E-19 | 1.00E-17 |
| RP11-467K18.2 | -4.012039207 | 3.337175984 | 1.36E-19 | 1.15E-17 |
| RP11-33A14.1 | -3.921536688 | 3.222247912 | 1.45E-19 | 1.20E-17 |
| RP1-269M15.3 | -4.504426977 | 6.08490469 | 1.79E-19 | 1.46E-17 |
| RP4-621B10.8 | -2.827307853 | 6.285620447 | 3.15E-19 | 2.54E-17 |
| RP11-321E2.3 | -4.798147245 | 4.022766269 | 3.86E-19 | 3.08E-17 |
| CTB-152G17.6 | -2.167674313 | 6.794608353 | 8.21E-19 | 6.47E-17 |
| RP11-173C1.1 | -3.181095223 | 4.865233198 | 8.95E-19 | 6.97E-17 |
| LINC00936 | -2.300211134 | 8.462868163 | 9.92E-19 | 7.62E-17 |
| RP11-10E18.7 | -2.649089714 | 4.557605895 | 1.63E-18 | 1.24E-16 |
| KRTAP5-AS1 | -3.621465246 | 5.813965184 | 2.06E-18 | 1.55E-16 |
| RP11-317N8.3 | -3.349369916 | 3.133922951 | 2.27E-18 | 1.68E-16 |
| CTD-2562J17.4 | -3.703771891 | 3.602777329 | 2.58E-18 | 1.89E-16 |
| RP11-662M24.2 | -4.237311834 | 2.702225078 | 2.70E-18 | 1.96E-16 |
| CTD-2020K17.3 | -2.7357512 | 6.035266854 | 2.87E-18 | 2.06E-16 |
| RP11-333I13.1 | -2.515303665 | 4.650012566 | 2.95E-18 | 2.09E-16 |
| RAPGEF4-AS1 | -3.227619253 | 4.521007965 | 3.90E-18 | 2.73E-16 |
| RP11-728G15.1 | -4.331439479 | 4.55819407 | 4.36E-18 | 3.02E-16 |
| KB-1517D11.4 | -4.812358408 | 6.196337639 | 4.48E-18 | 3.07E-16 |
| LINC01511 | -4.794591344 | 4.74577128 | 4.55E-18 | 3.08E-16 |
| CTD-2235C13.3 | -2.677747123 | 4.227462668 | 5.34E-18 | 3.58E-16 |
| RP4-784A16.5 | -3.322236758 | 3.361897418 | 5.95E-18 | 3.95E-16 |
| PPP1R26-AS1 | -2.338248267 | 6.020246791 | 6.15E-18 | 4.04E-16 |
| AC091878.1 | -4.088912146 | 5.000999042 | 6.84E-18 | 4.45E-16 |
| MAPT-IT1 | -3.531360752 | 5.494562084 | 1.16E-17 | 7.44E-16 |
| LINC01260 | -3.184202041 | 5.204456082 | 1.18E-17 | 7.49E-16 |
| RP11-345N11.1 | -3.62311654 | 3.212642726 | 2.39E-17 | 1.49E-15 |
| CPEB1-AS1 | -2.732994513 | 4.598206524 | 2.51E-17 | 1.55E-15 |
| RP11-286B14.1 | -4.740789634 | 6.785075136 | 3.37E-17 | 2.06E-15 |
| AP002954.3 | -2.922176557 | 5.305592572 | 3.43E-17 | 2.08E-15 |
| STXBP5-AS1 | -2.612458194 | 6.688024873 | 5.40E-17 | 3.24E-15 |
| DLGAP1-AS4 | -3.97321958 | 5.733037874 | 5.69E-17 | 3.38E-15 |
| RP11-446H18.5 | -2.384700102 | 4.437200711 | 1.31E-16 | 7.69E-15 |
| RP11-684N3.1 | -4.241877295 | 4.495285032 | 2.00E-16 | 1.16E-14 |
| RP11-81H3.2 | -4.361163336 | 3.675624517 | 2.31E-16 | 1.33E-14 |
| CTA-221G9.12 | -2.81813256 | 4.432171791 | 2.36E-16 | 1.35E-14 |
| RP11-346C4.3 | -3.647804722 | 5.15734651 | 3.39E-16 | 1.92E-14 |
| LINC00202-1 | -3.13366107 | 5.559073254 | 4.21E-16 | 2.37E-14 |
| C22orf24 | -2.415383655 | 4.579470588 | 4.73E-16 | 2.64E-14 |
| CTD-2527I21.5 | -3.27011965 | 4.400641645 | 4.85E-16 | 2.68E-14 |
| RP11-770J1.4 | -2.442136608 | 4.34313024 | 5.29E-16 | 2.90E-14 |
| RP11-101E7.2 | -2.220961989 | 4.11537911 | 5.76E-16 | 3.13E-14 |
| RP11-95O2.1 | -3.621511406 | 4.484772175 | 6.12E-16 | 3.30E-14 |
| AC000403.4 | -2.651588536 | 6.310825896 | 7.44E-16 | 3.97E-14 |
| RP11-298E9.6 | -4.039528747 | 3.15018234 | 8.64E-16 | 4.57E-14 |
| RP6-191P20.4 | -4.598907929 | 4.412581755 | 8.85E-16 | 4.65E-14 |
| CASC18 | -3.415280185 | 4.946556984 | 9.11E-16 | 4.74E-14 |
| LINC00982 | -2.890967929 | 8.530715057 | 9.24E-16 | 4.78E-14 |
| RP11-158J3.2 | -4.113259639 | 3.61042906 | 1.07E-15 | 5.48E-14 |
| RNF144A-AS1 | -3.657491823 | 6.890117762 | 1.33E-15 | 6.77E-14 |
| RP11-13A1.1 | -2.847729851 | 5.162612415 | 1.35E-15 | 6.78E-14 |
| RP11-586D19.1 | -3.782658067 | 5.905889837 | 1.38E-15 | 6.91E-14 |
| RP1-240B8.3 | -4.256700178 | 3.18393321 | 1.59E-15 | 7.90E-14 |
| CTD-2292M16.8 | -2.071620061 | 6.389899912 | 1.84E-15 | 9.08E-14 |
| RP11-50D16.4 | -2.666111577 | 4.216789787 | 2.07E-15 | 1.01E-13 |
| RP11-29G8.3 | -2.108460588 | 5.371400066 | 2.30E-15 | 1.12E-13 |
| HAR1A | -3.55122986 | 5.915488709 | 2.45E-15 | 1.18E-13 |
| AC091729.8 | -3.171726875 | 3.043380186 | 2.65E-15 | 1.27E-13 |
| RASGRF2-AS1 | -3.145615814 | 3.818327332 | 2.85E-15 | 1.35E-13 |
| KB-1742H10.3 | -2.447450451 | 5.702547613 | 3.72E-15 | 1.75E-13 |
| RP13-977J11.3 | -3.671687076 | 3.684193637 | 4.01E-15 | 1.87E-13 |
| RP11-799B12.4 | -2.052502992 | 5.054690526 | 4.23E-15 | 1.96E-13 |
| RP11-845M18.6 | -4.199896183 | 3.171515025 | 5.20E-15 | 2.39E-13 |
| AC012074.2 | -2.302892761 | 5.064917489 | 7.69E-15 | 3.51E-13 |
| CTD-2527I21.4 | -4.07830442 | 3.73552988 | 8.10E-15 | 3.67E-13 |
| CTD-2228A4.1 | -4.261876228 | 3.258901664 | 1.02E-14 | 4.57E-13 |
| HTR5A-AS1 | -4.477553824 | 5.927195185 | 1.58E-14 | 7.01E-13 |
| THSD4-AS1 | -4.326966666 | 4.167249574 | 1.73E-14 | 7.64E-13 |
| TMEM191A | -2.434885136 | 5.702801599 | 2.10E-14 | 9.14E-13 |
| RP11-566K19.6 | -3.446183097 | 6.635691166 | 2.14E-14 | 9.22E-13 |
| RP11-271F18.4 | -4.374426618 | 3.80541629 | 2.50E-14 | 1.07E-12 |
| RP11-806L2.2 | -2.709794521 | 4.927537005 | 3.05E-14 | 1.30E-12 |
| RP11-388P9.2 | -3.079671873 | 3.014924488 | 3.38E-14 | 1.43E-12 |
| CTC-525D6.1 | -4.000967191 | 5.649923522 | 3.70E-14 | 1.55E-12 |
| RP11-455F5.5 | -2.213278886 | 5.007767313 | 3.85E-14 | 1.61E-12 |
| CTD-2023N9.3 | -4.349954158 | 5.233376382 | 4.03E-14 | 1.67E-12 |
| GS1-72M22.1 | -4.090873428 | 6.119458941 | 4.60E-14 | 1.90E-12 |
| LINC01123 | -4.227993942 | 4.593249346 | 5.28E-14 | 2.16E-12 |
| RP11-1263C18.1 | -3.56110369 | 6.239360914 | 5.65E-14 | 2.30E-12 |
| MIR600HG | -2.423446817 | 9.293632977 | 6.40E-14 | 2.58E-12 |
| RP5-1119A7.17 | -4.145187006 | 7.546824426 | 6.43E-14 | 2.58E-12 |
| RP11-284N8.3 | -3.04364795 | 7.857305238 | 7.20E-14 | 2.87E-12 |
| RP3-425C14.4 | -2.006640713 | 9.665382592 | 1.18E-13 | 4.63E-12 |
| LINC01484 | -3.209961784 | 3.224600776 | 1.34E-13 | 5.17E-12 |
| AC011288.2 | -4.301917947 | 3.604936937 | 1.50E-13 | 5.69E-12 |
| CTD-3193O13.1 | -2.327521052 | 3.598369174 | 1.50E-13 | 5.69E-12 |
| TMEM72-AS1 | -2.173916768 | 5.203066897 | 1.88E-13 | 7.09E-12 |
| RP11-762L8.6 | -2.018275327 | 7.074285226 | 2.05E-13 | 7.68E-12 |
| RP11-234K24.3 | -3.02169809 | 5.507646331 | 2.12E-13 | 7.92E-12 |
| LLNLF-187D8.1 | -2.08737383 | 8.382867252 | 2.16E-13 | 7.99E-12 |
| RP11-119F19.5 | -2.046590028 | 5.579822866 | 2.17E-13 | 7.99E-12 |
| RP11-416N2.4 | -2.9432124 | 6.062296201 | 2.19E-13 | 8.04E-12 |
| RP1-63G5.5 | -3.566043476 | 5.450740937 | 2.35E-13 | 8.56E-12 |
| RP11-285F16.1 | -2.43986641 | 5.101843457 | 2.55E-13 | 9.23E-12 |
| RP11-547D24.1 | -3.128738416 | 4.802516918 | 2.66E-13 | 9.56E-12 |
| RP11-298J23.8 | -2.897349615 | 2.911559742 | 3.05E-13 | 1.09E-11 |
| AC141928.1 | -3.625506667 | 6.083657865 | 3.19E-13 | 1.13E-11 |
| RP11-863P13.5 | -3.625970352 | 4.115394674 | 3.30E-13 | 1.16E-11 |
| RP13-895J2.7 | -4.154580142 | 4.052980391 | 3.83E-13 | 1.34E-11 |
| RP11-379F4.4 | -2.261846593 | 6.446122567 | 4.70E-13 | 1.63E-11 |
| RP11-677M14.2 | -3.207529179 | 3.002241503 | 4.72E-13 | 1.63E-11 |
| RP11-433J8.1 | -3.686134683 | 5.627714711 | 5.09E-13 | 1.75E-11 |
| CTD-3193K9.3 | -2.514648637 | 4.420362719 | 7.65E-13 | 2.61E-11 |
| MIR124-2HG | -3.680206811 | 7.432306225 | 1.77E-12 | 5.92E-11 |
| AC011516.2 | -4.49722261 | 3.985494747 | 1.93E-12 | 6.44E-11 |
| RP11-463C8.7 | -3.053000453 | 4.835393639 | 2.42E-12 | 7.96E-11 |
| TNK2-AS1 | -2.766396249 | 4.559309755 | 2.42E-12 | 7.96E-11 |
| TUNAR | -3.775024724 | 7.20059124 | 2.76E-12 | 9.02E-11 |
| TMEM108-AS1 | -3.612415638 | 3.476428359 | 2.94E-12 | 9.56E-11 |
| RP11-710C12.1 | -3.081145291 | 6.917149654 | 3.04E-12 | 9.84E-11 |
| RP3-395M20.3 | -3.725998747 | 3.24013927 | 3.22E-12 | 1.04E-10 |
| RP11-385G16.1 | -3.197776683 | 4.62019862 | 3.31E-12 | 1.06E-10 |
| RP11-588K22.2 | -2.3789119 | 8.888915997 | 3.92E-12 | 1.24E-10 |
| AC118754.4 | -2.852887615 | 2.703813424 | 4.00E-12 | 1.25E-10 |
| RP11-508N22.12 | -2.150381312 | 6.047283297 | 4.08E-12 | 1.27E-10 |
| RP11-573G6.6 | -2.280070387 | 4.236216194 | 5.07E-12 | 1.58E-10 |
| AC009014.3 | -3.642963471 | 5.101667095 | 5.55E-12 | 1.72E-10 |
| RP11-826N14.2 | -4.194852302 | 4.631180212 | 6.11E-12 | 1.88E-10 |
| LINC01554 | -2.105639563 | 6.126149342 | 7.30E-12 | 2.22E-10 |
| RP11-953B20.1 | -4.091539529 | 4.406221964 | 8.71E-12 | 2.64E-10 |
| RP11-140A10.3 | -2.783206465 | 3.452256406 | 9.17E-12 | 2.76E-10 |
| RP11-1134I14.8 | -2.081872169 | 7.998369507 | 9.48E-12 | 2.85E-10 |
| AC103563.8 | -2.923579494 | 4.074675826 | 1.01E-11 | 3.00E-10 |
| CTD-2001J20.1 | -3.250707889 | 5.687788786 | 1.06E-11 | 3.10E-10 |
| RP11-731J8.2 | -3.033035564 | 10.16293644 | 1.35E-11 | 3.94E-10 |
| PWAR5 | -2.607932885 | 6.303955201 | 1.60E-11 | 4.64E-10 |
| LINC01202 | -4.733386127 | 4.298716068 | 1.62E-11 | 4.67E-10 |
| RP3-395M20.2 | -3.65748322 | 3.19352462 | 1.62E-11 | 4.67E-10 |
| RP11-197M22.2 | -2.347707599 | 3.548742911 | 1.90E-11 | 5.41E-10 |
| RP4-735C1.4 | -2.662436325 | 3.679174109 | 1.91E-11 | 5.43E-10 |
| RP11-384O8.1 | -2.858266016 | 6.824336255 | 2.01E-11 | 5.67E-10 |
| RP11-897M7.4 | -2.920635552 | 2.885443548 | 2.81E-11 | 7.90E-10 |
| LINC01099 | -3.061690252 | 2.945030028 | 3.22E-11 | 9.03E-10 |
| RP13-616I3.1 | -2.349062513 | 5.20157959 | 3.35E-11 | 9.34E-10 |
| RP11-301G7.1 | -3.615869472 | 3.632286878 | 3.38E-11 | 9.38E-10 |
| RP11-946L20.2 | -3.762814844 | 4.07278314 | 3.40E-11 | 9.39E-10 |
| RP11-701H24.5 | -2.959997175 | 3.175159785 | 3.64E-11 | 1.00E-09 |
| FAM201A | -3.2966646 | 5.622684338 | 4.26E-11 | 1.17E-09 |
| UMODL1-AS1 | -3.649110608 | 4.695615904 | 4.59E-11 | 1.25E-09 |
| AC137932.6 | -2.1563489 | 4.726300526 | 4.70E-11 | 1.28E-09 |
| RP11-66B24.4 | -3.173090895 | 7.01934884 | 4.93E-11 | 1.33E-09 |
| LINC00507 | -4.48819708 | 6.231727044 | 4.98E-11 | 1.34E-09 |
| RP11-482M8.3 | -3.774729693 | 5.517164506 | 5.62E-11 | 1.51E-09 |
| DGCR5 | -2.217687841 | 9.871350539 | 6.15E-11 | 1.64E-09 |
| MIR7-3HG | -3.481838022 | 6.920499657 | 6.66E-11 | 1.76E-09 |
| RP11-262I2.2 | -3.465727891 | 3.091722338 | 7.76E-11 | 2.03E-09 |
| RP11-430B1.2 | -2.036751014 | 6.032594764 | 8.46E-11 | 2.20E-09 |
| CTC-344H19.4 | -2.368217423 | 3.792542612 | 8.84E-11 | 2.29E-09 |
| AC067969.2 | -3.454917666 | 3.405338712 | 9.87E-11 | 2.54E-09 |
| C17orf102 | -2.864255891 | 5.667184076 | 1.08E-10 | 2.76E-09 |
| RP11-1406H17.1 | -3.451118627 | 3.739901927 | 1.13E-10 | 2.87E-09 |
| RP3-355L5.5 | -2.309395304 | 4.311034384 | 1.20E-10 | 3.04E-09 |
| CTD-2562J17.7 | -2.534685053 | 3.32913955 | 1.28E-10 | 3.21E-09 |
| RP11-127B20.2 | -2.108595078 | 6.995607721 | 1.56E-10 | 3.90E-09 |
| MIAT | -2.797880084 | 11.41069651 | 2.49E-10 | 6.09E-09 |
| MIR4500HG | -3.00722313 | 5.185019058 | 2.83E-10 | 6.88E-09 |
| PP14571 | -2.354958238 | 6.007369688 | 2.84E-10 | 6.88E-09 |
| RP11-713C5.1 | -3.454990275 | 8.350186881 | 2.98E-10 | 7.20E-09 |
| RP11-286N22.10 | -3.07010315 | 3.664488761 | 3.26E-10 | 7.85E-09 |
| CTC-332L22.1 | -2.053926511 | 4.983039572 | 3.58E-10 | 8.55E-09 |
| RP6-65G23.3 | -2.646938484 | 5.993791201 | 4.25E-10 | 1.00E-08 |
| RP11-881M11.4 | -2.47486258 | 5.006744133 | 4.37E-10 | 1.03E-08 |
| EMX2OS | -2.637135615 | 9.918626868 | 4.60E-10 | 1.08E-08 |
| AC005330.2 | -2.040795868 | 7.706480806 | 5.46E-10 | 1.27E-08 |
| AP001469.5 | -2.079680109 | 3.627218913 | 5.49E-10 | 1.27E-08 |
| RP11-121G22.3 | -2.412117967 | 4.233596739 | 5.59E-10 | 1.29E-08 |
| RP13-895J2.8 | -3.751461044 | 3.686388748 | 6.96E-10 | 1.59E-08 |
| RP11-986G18.2 | -2.204252306 | 3.668024597 | 7.47E-10 | 1.70E-08 |
| RP11-760H22.2 | -2.10272956 | 6.586960561 | 9.62E-10 | 2.15E-08 |
| RP11-423H2.3 | -2.278663369 | 5.381919652 | 1.16E-09 | 2.57E-08 |
| RP5-1039K5.16 | -3.072768569 | 4.008977864 | 1.21E-09 | 2.65E-08 |
| RP11-830F9.7 | -2.655885013 | 3.175741021 | 1.74E-09 | 3.77E-08 |
| RP11-314B1.2 | -3.267229514 | 6.3203744 | 1.81E-09 | 3.87E-08 |
| RP11-247C2.2 | -3.439848955 | 5.75050135 | 2.02E-09 | 4.28E-08 |
| AC003003.5 | -2.681569739 | 5.306452429 | 2.44E-09 | 5.11E-08 |
| RP11-131L12.3 | -2.106435403 | 4.948195875 | 2.75E-09 | 5.70E-08 |
| AC097468.7 | -2.615076266 | 3.081823668 | 2.83E-09 | 5.83E-08 |
| RP11-98D18.15 | -2.747440601 | 3.473772273 | 2.95E-09 | 6.02E-08 |
| IL12A-AS1 | -2.090227111 | 4.601247173 | 3.24E-09 | 6.59E-08 |
| C3orf67-AS1 | -3.705004173 | 3.87183948 | 3.50E-09 | 7.05E-08 |
| AC018647.3 | -2.146276171 | 8.948471963 | 3.51E-09 | 7.05E-08 |
| LINC00599 | -3.163223485 | 8.820488761 | 4.04E-09 | 8.08E-08 |
| RP11-407G23.7 | -2.016499398 | 4.93133912 | 4.21E-09 | 8.34E-08 |
| CTC-529P8.1 | -2.488609487 | 3.833610642 | 5.49E-09 | 1.07E-07 |
| RP4-564M11.2 | -3.036468091 | 3.290955814 | 5.63E-09 | 1.09E-07 |
| CTA-250D10.19 | -2.498580085 | 3.064131384 | 5.90E-09 | 1.14E-07 |
| LINC00320 | -2.852528478 | 8.685317332 | 7.22E-09 | 1.38E-07 |
| FAM95C | -3.316041414 | 5.026339393 | 7.82E-09 | 1.48E-07 |
| AL450992.2 | -2.619976954 | 6.280531041 | 9.94E-09 | 1.87E-07 |
| RP11-273G15.2 | -2.250288644 | 6.772910236 | 1.18E-08 | 2.20E-07 |
| CTA-268H5.14 | -2.079505125 | 4.080064881 | 1.23E-08 | 2.27E-07 |
| LINC00951 | -2.286010142 | 4.462350587 | 1.43E-08 | 2.60E-07 |
| RP13-514E23.2 | -2.39043928 | 3.361905111 | 1.62E-08 | 2.92E-07 |
| LINC01018 | -2.70989618 | 6.599099764 | 1.83E-08 | 3.27E-07 |
| AC012593.1 | -3.208308083 | 3.456841421 | 1.83E-08 | 3.27E-07 |
| GPR123-AS1 | -2.640915038 | 6.855420793 | 2.50E-08 | 4.37E-07 |
| RP11-131L12.4 | -2.032291207 | 4.84083995 | 2.60E-08 | 4.51E-07 |
| RP11-830F9.5 | -2.572740989 | 3.388718839 | 2.63E-08 | 4.55E-07 |
| RP11-496D24.2 | -2.354448116 | 4.80965639 | 2.73E-08 | 4.71E-07 |
| KCNC4-AS1 | -2.131619999 | 3.963812681 | 2.82E-08 | 4.84E-07 |
| RP11-38L15.3 | -2.041694972 | 4.99419033 | 3.14E-08 | 5.36E-07 |
| SAPCD1-AS1 | -2.526831479 | 3.099376381 | 3.77E-08 | 6.39E-07 |
| RP11-982M15.6 | -2.824967443 | 4.167817483 | 3.81E-08 | 6.41E-07 |
| AC000095.11 | -2.432217223 | 4.371654142 | 4.05E-08 | 6.77E-07 |
| RP11-66B24.1 | -2.595305057 | 3.610096887 | 5.18E-08 | 8.62E-07 |
| RP11-85O21.2 | -2.614753188 | 5.579414405 | 5.43E-08 | 8.98E-07 |
| LINC01336 | -2.1242115 | 3.273095716 | 5.52E-08 | 9.11E-07 |
| AC093590.1 | -2.67775594 | 4.294979573 | 7.57E-08 | 1.21E-06 |
| RP13-554M15.8 | -2.060759792 | 3.268007734 | 7.73E-08 | 1.23E-06 |
| AC131056.3 | -3.002643692 | 3.776427212 | 8.37E-08 | 1.32E-06 |
| RP13-870H17.3 | -2.424018909 | 4.279927029 | 8.99E-08 | 1.41E-06 |
| LINC00622 | -2.157941888 | 5.616608472 | 9.76E-08 | 1.50E-06 |
| RP11-44N21.1 | -2.462705621 | 4.662676347 | 1.00E-07 | 1.53E-06 |
| CTA-228A9.3 | -2.012624854 | 6.464629349 | 1.06E-07 | 1.61E-06 |
| AC092667.2 | -2.27940991 | 4.745288379 | 1.09E-07 | 1.65E-06 |
| CTB-113P19.4 | -2.470667483 | 4.217944313 | 1.16E-07 | 1.74E-06 |
| RP11-127I20.5 | -2.436983577 | 3.492623315 | 1.25E-07 | 1.87E-06 |
| RP11-700H6.4 | -2.328440142 | 3.109310826 | 1.28E-07 | 1.91E-06 |
| CTD-2380F24.1 | -2.463231394 | 5.133890631 | 1.32E-07 | 1.97E-06 |
| RP11-527D7.1 | -2.551420699 | 5.719579589 | 1.42E-07 | 2.09E-06 |
| HCG2040054 | -2.364620618 | 2.812015814 | 1.51E-07 | 2.22E-06 |
| RP3-395M20.12 | -2.037217931 | 5.58008393 | 1.64E-07 | 2.39E-06 |
| RP11-106M7.1 | -2.071169967 | 5.582817051 | 1.85E-07 | 2.65E-06 |
| RP11-88H12.2 | -2.109459441 | 5.422223979 | 1.90E-07 | 2.71E-06 |
| MIR137HG | -2.705604578 | 6.275362728 | 1.96E-07 | 2.78E-06 |
| RP11-299G20.5 | -2.311854283 | 5.944923538 | 2.90E-07 | 4.01E-06 |
| RP11-181B11.1 | -2.121769119 | 3.262854253 | 3.32E-07 | 4.54E-06 |
| CTD-2547L24.3 | -2.028213285 | 4.344847794 | 3.57E-07 | 4.84E-06 |
| DKFZp434J0226 | -2.86966208 | 5.237568909 | 3.90E-07 | 5.24E-06 |
| LINC00642 | -2.244506397 | 3.727385254 | 4.04E-07 | 5.41E-06 |
| RP11-379L18.1 | -2.118052773 | 4.525216402 | 4.28E-07 | 5.70E-06 |
| RP11-21A7A.2 | -2.31153821 | 4.074139274 | 4.33E-07 | 5.74E-06 |
| CTB-78F1.1 | -3.326683944 | 4.009220917 | 4.60E-07 | 6.07E-06 |
| RP11-437J2.4 | -2.569276617 | 3.022778132 | 6.09E-07 | 7.90E-06 |
| RP5-827C21.6 | -2.848938784 | 4.005772947 | 6.64E-07 | 8.52E-06 |
| DPP10-AS1 | -3.010160061 | 6.024627267 | 6.80E-07 | 8.72E-06 |
| CTD-2062F14.3 | -2.197669084 | 3.18094115 | 7.36E-07 | 9.38E-06 |
| AC007163.3 | -2.932482193 | 5.173866519 | 7.36E-07 | 9.38E-06 |
| C11orf39 | -2.584729321 | 6.059281902 | 7.77E-07 | 9.84E-06 |
| LMF1-AS1 | -2.269366557 | 4.544875226 | 8.13E-07 | 1.02E-05 |
| CTD-2334D19.1 | -2.001081882 | 4.138974059 | 8.57E-07 | 1.07E-05 |
| LINC01122 | -2.131431003 | 6.449420526 | 1.02E-06 | 1.24E-05 |
| RP11-338K13.1 | -2.763751416 | 3.512114035 | 1.12E-06 | 1.35E-05 |
| RP11-561B11.6 | -3.0182075 | 3.329519856 | 1.18E-06 | 1.42E-05 |
| RP11-353N14.4 | -2.047811994 | 4.746301591 | 1.18E-06 | 1.42E-05 |
| RP11-54O7.18 | -2.137918756 | 3.423100745 | 1.54E-06 | 1.83E-05 |
| RP11-1C8.4 | -2.950451576 | 4.133073221 | 1.82E-06 | 2.11E-05 |
| RP11-573D15.2 | -2.02405643 | 4.783826851 | 1.90E-06 | 2.19E-05 |
| RP11-74E22.8 | -2.141995829 | 8.585542627 | 1.93E-06 | 2.22E-05 |
| RP11-286E11.1 | -2.416219293 | 5.721494876 | 2.32E-06 | 2.63E-05 |
| RP11-809C18.3 | -2.737629686 | 7.121691783 | 2.54E-06 | 2.85E-05 |
| LINC01574 | -2.446871796 | 3.911848049 | 2.85E-06 | 3.15E-05 |
| RP11-272L13.3 | -2.619122967 | 3.517856305 | 2.93E-06 | 3.22E-05 |
| HPN-AS1 | -2.801795181 | 6.017101117 | 3.02E-06 | 3.31E-05 |
| RP11-61I13.3 | -2.093221589 | 8.130443105 | 3.09E-06 | 3.37E-05 |
| RP4-655C5.9 | -2.190037091 | 3.519998323 | 3.15E-06 | 3.42E-05 |
| ADARB2-AS1 | -3.404724649 | 4.489915526 | 3.18E-06 | 3.45E-05 |
| RP11-245J24.1 | -3.046290606 | 3.954175606 | 3.28E-06 | 3.55E-05 |
| RP11-153F1.1 | -2.140779192 | 3.307257159 | 4.07E-06 | 4.26E-05 |
| PAQR9-AS1 | -2.149594993 | 5.234277492 | 6.23E-06 | 6.25E-05 |
| RORB-AS1 | -2.21196724 | 5.160857035 | 6.60E-06 | 6.61E-05 |
| RP11-109I13.2 | -2.237296643 | 3.23364978 | 7.02E-06 | 6.98E-05 |
| CTC-338M12.1 | -2.131766014 | 3.034140847 | 7.27E-06 | 7.19E-05 |
| RP11-369E15.3 | -3.129848243 | 4.189342282 | 7.92E-06 | 7.78E-05 |
| UNC5B-AS1 | -2.014566855 | 4.261644628 | 7.95E-06 | 7.79E-05 |
| RP1-46F2.3 | -3.369386795 | 5.011737872 | 8.16E-06 | 7.99E-05 |
| RP13-895J2.2 | -2.980662081 | 3.49162148 | 9.04E-06 | 8.71E-05 |
| LA16c-329F2.1 | -2.794169216 | 3.715673533 | 9.19E-06 | 8.83E-05 |
| LINC01105 | -2.155657733 | 10.21262167 | 9.26E-06 | 8.88E-05 |
| RP11-798K3.2 | -2.603334135 | 4.092984623 | 1.04E-05 | 9.83E-05 |
| CTD-2269F5.1 | -2.346679993 | 4.807696733 | 1.23E-05 | 0.000114388 |
| PACRG-AS1 | -2.161977908 | 4.84370969 | 1.25E-05 | 0.000115481 |
| CTB-12A17.2 | -2.156148773 | 5.591580923 | 1.32E-05 | 0.00012145 |
| RP11-64P14.7 | -2.530466722 | 3.261546575 | 1.33E-05 | 0.000121828 |
| RP11-1055B8.3 | -2.326483479 | 7.36740187 | 1.40E-05 | 0.000126903 |
| CTC-525D6.2 | -2.771275854 | 3.536252127 | 1.40E-05 | 0.000127113 |
| RP11-13K12.2 | -2.388512175 | 4.788911627 | 1.59E-05 | 0.00014244 |
| RP11-173A16.1 | -2.036386625 | 3.149688284 | 2.32E-05 | 0.000199056 |
| LINC00463 | -2.215849696 | 5.252060468 | 2.47E-05 | 0.000210124 |
| RP11-377G16.2 | -2.41618066 | 3.032170135 | 2.75E-05 | 0.0002321 |
| RP11-417L19.2 | -2.192494072 | 3.848748902 | 3.76E-05 | 0.000304545 |
| RP1-223B1.1 | -2.100325639 | 5.833330158 | 4.32E-05 | 0.000344428 |
| RP11-527N22.2 | -2.560911553 | 4.521916056 | 4.38E-05 | 0.000348876 |
| HAR1B | -2.526328145 | 4.204581411 | 4.39E-05 | 0.000349822 |
| RP11-495O11.1 | -2.037810208 | 3.547550746 | 4.74E-05 | 0.00037137 |
| RP11-150O12.1 | -2.464094268 | 6.756573671 | 4.95E-05 | 0.000385681 |
| RP4-719C8.1 | -2.501529357 | 4.802291557 | 5.44E-05 | 0.000419806 |
| RP1-293L6.1 | -2.774581495 | 4.28649551 | 6.39E-05 | 0.000481629 |
| RP11-1C8.7 | -2.632865629 | 3.549506761 | 6.85E-05 | 0.000510795 |
| VSTM2A-OT1 | -3.184046827 | 5.790357302 | 7.15E-05 | 0.000524391 |
| RP4-630C24.3 | -2.462239928 | 4.446625409 | 8.64E-05 | 0.000616924 |
| RP11-439M11.1 | -2.732646702 | 3.777717284 | 9.58E-05 | 0.000669951 |
| RP11-1029J19.4 | -2.05552833 | 4.257497294 | 0.000108499 | 0.0007508 |
| RP11-662G23.1 | -2.370090958 | 3.959214931 | 0.000112316 | 0.000772129 |
| LINC00632 | -2.164060852 | 7.137900053 | 0.000127851 | 0.000860164 |
| RP11-227D2.3 | -2.21755762 | 2.623842599 | 0.000128363 | 0.000862684 |
| RP13-977J11.8 | -2.12606571 | 2.839761187 | 0.000130083 | 0.000873313 |
| KCNQ1DN | -2.129993954 | 4.636671342 | 0.000142147 | 0.000943257 |
