# Supplementary figures and images for: Long noncoding RNA UNC5B-AS1 suppresses cell proliferation by sponging miR-24-3p in glioblastoma multiforme
Source: BMC Med Genomics. 2024 Apr 9;17:83. doi: 10.1186/s12920-024-01851-5 (PMC11003007; doi:10.1186/s12920-024-01851-5)

**A.Figure 1.** The research process for this study.


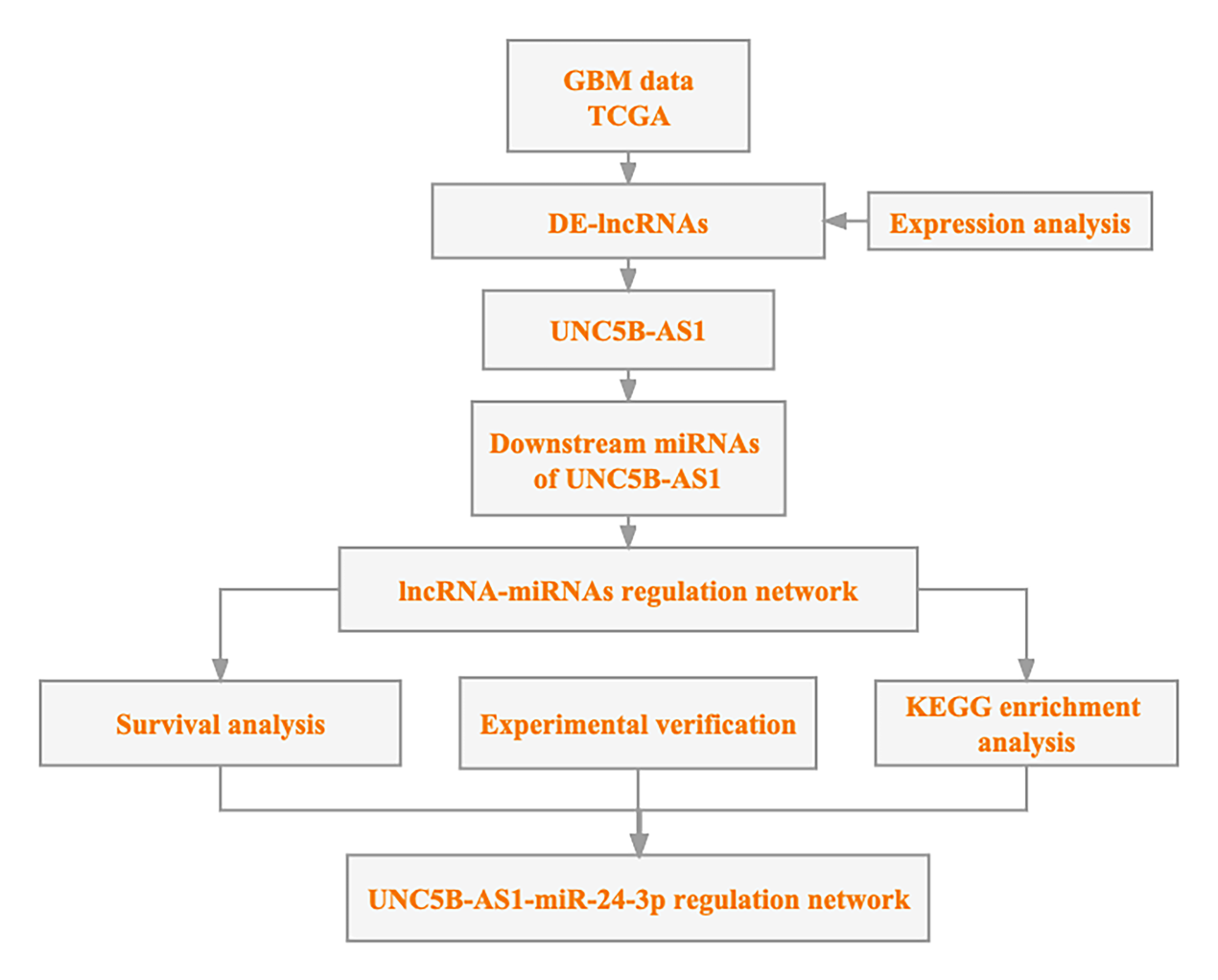

Supplement: Supplementary file 1 — Supplementary Material 1. [file 12920_2024_1851_MOESM1_ESM.zip › Additional file 1/A.Figure 1.docx]
